# Assessment of Cognitive Function with Sleep Spindle Characteristics in Adults with Epilepsy

**DOI:** 10.1155/2023/7768980

**Published:** 2023-04-17

**Authors:** Yajin Huang, Yaqing Liu, Wenjun Song, Yanjun Liu, Xiaoqian Wang, Juping Han, Jiang Ye, Hongmei Han, Li Wang, Juan Li, Tiancheng Wang

**Affiliations:** ^1^The Second Clinical Medical College, Lanzhou University/Department of Neurology, Epilepsy Center, Lanzhou University Second Hospital, Lanzhou University, Lanzhou 730000, China; ^2^Department of Neurology, Epilepsy Center, Lanzhou University Second Hospital, Lanzhou University, Lanzhou 730000, China

## Abstract

**Objective:**

Epilepsy may cause chronic cognitive impairment by disturbing sleep plasticity. Sleep spindles play a crucial role in sleep maintenance and brain plasticity. This study explored the relationship between cognition and spindle characteristics in adult epilepsy.

**Methods:**

Participants underwent one-night sleep electroencephalogram recording and neuropsychological tests on the same day. Spindle characteristics during N2 sleep were extracted using a learning-based system for sleep staging and an automated spindle detection algorithm. We investigated the difference between cognitive subgroups in spindle characteristics. Multiple linear regressions were applied to analyze associations between cognition and spindle characteristics.

**Results:**

Compared with no/mild cognitive impairment, epilepsy patients who developed severe cognitive impairment had lower sleep spindle density, the differences mainly distributed in central, occipital, parietal, middle temporal, and posterior temporal (*P* < 0.05), and had relatively long spindle duration in occipital and posterior temporal (*P* < 0.05). Mini-Mental State Examination (MMSE) was associated with spindle density (pars triangularis of the inferior frontal gyrus (IFGtri): *β* = 0.253, *P* = 0.015, and *P*.adjust = 0.074) and spindle duration (IFGtri: *β* = −0.262, *P* = 0.004, and *P*.adjust = 0.030). Montreal Cognitive Assessment (MoCA) was associated with spindle duration (IFGtri: *β* = −0.246, *P* = 0.010, and *P*.adjust = 0.055). Executive Index Score (MoCA-EIS) was associated with spindle density (IFGtri: *β* = 0.238, *P* = 0.019, and *P*.adjust = 0.087; parietal: *β* = 0.227, *P* = 0.017, and *P*.adjust = 0.082) and spindle duration (parietal: *β* = −0.230, *P* = 0.013, and *P*.adjust = 0.065). Attention Index Score (MoCA-AIS) was associated with spindle duration (IFGtri: *β* = −0.233, *P* = 0.017, and *P*.adjust = 0.081).

**Conclusions:**

The findings suggested that the altered spindle activity in epilepsy with severe cognitive impairment, the associations between the global cognitive status of adult epilepsy and spindle characteristics, and specific cognitive domains may relate to spindle characteristics in particular brain regions.

## 1. Introduction

Epilepsy is one of the most common neurological conditions, characterized by chronic, recurrent, and unprovoked seizures. Cognitive comorbidity is prevalent in epilepsy, and approximately 70-80% of chronic epilepsy has cognitive decline [1]. The clinical signs are memory decay, attention, information-processing speed, and executive function lesions, with adverse impact on the quality of life. Unfortunately, early diagnosis of cognitive impairment still lacks reliable biomarkers. Besides symptomatic treatment, there is no effective therapy available.

Several studies have shown a close link between epilepsy, nonrapid eye movement (NREM) sleep, and neuroplasticity [2–4]. NREM sleep promotes seizure activity, whereas rapid eye movement (REM) sleep decreases seizure susceptibility [5]. NREM sleep participates in multiple essential brain functions, such as synaptic homeostasis [6], brain plasticity [3, 7], memory consolidation [8], and learning [9]. Accumulating evidence reveals brain dysfunction in epilepsy [10, 11], which involves sleep plasticity-related structures, such as thalamocortical and frontal-hippocampal systems [4, 10]. Epilepsy may cause chronic cognitive impairment by disturbing sleep plasticity [2, 4].

Sleep spindles are a hallmark of NREM sleep stage 2 (N2), with relatively brief duration (0.5-3 s) and fusiform [12]. Sleep spindles are initiated by the thalamic reticular nucleus (TRN) and regulated by thalamic-reticular and thalamic-cortical circuits, which play an essential role in sleep maintenance, synaptic plasticity, and memory formation [13]. Sleep spindle characteristics change throughout the lifespan. Sleep spindles gradually maturate from childhood through late adolescence and vary with age [14, 15]. Brain reorganization during adolescence produces massive changes in sleep EEG, including the morphology and abundance of sleep spindles (e.g., frequency, amplitude, density, and duration) [14]. Further, the progressive decrease of sleep spindle density is considered a feature of normal aging [13, 16], and spindle amplitude also has proven correlative with cerebral atrophy [17]. These changes in the sleep spindle accompany brain development and aging [13, 16], and brain structure, such as the integrity of gray and white matter, gradually reduces likewise during normal aging [18]. Sleep spindle activity may reflect the maturation, late development, and interruption of thalamocortical regulatory mechanisms [13, 15, 16].

So far, there are relatively few reports on the relationship between sleep spindles and cognitive function in epilepsy patients. In one of these studies, children with benign epilepsy with centrotemporal spikes have a positive relationship between sleep spindle rate in centrotemporal regions and general intelligence, processing speed, and fine motor [19]. There is also a link between the sleep spindle, brain development, and age [13–17]. Spindle activity is widespread among cortical areas, whereas its spatiotemporal features are nonuniform and influenced by cortical locations [20–22]. Moreover, epilepsy is a heterogeneous disease, and the relationships between cognitive symptoms and sleep spindles in people with other types of epilepsy are as yet unclear. More types of evidence are needed to better understand the pathophysiology of seizures and cognitive dysfunction in epilepsy.

In the current study, we raised the hypothesis of whether a similar phenomenon could be observed in adults with epilepsy. We validated this hypothesis by investigating the cognitive function and N2 sleep spindle characteristics among fifty-seven adults with epilepsy from a tertiary epilepsy center. In this study, we introduced a neural network approach to process EEG data and obtain the results of sleep staging and spindle parameters. From the perspective of neuropsychology and neuroelectrophysiology, we aimed to identify the difference in spindle features among cognitive subgroups in epilepsy. Besides that, we also explored the relationship between spindle measures of different brain regions and specific neurocognitive deficits in adults with epilepsy, which may provide further insight into the neural circuits underlying such impairments. Importantly, there are currently no available validated biomarkers to identify cognitive risk or proven strategies to treat cognitive dysfunction in adults with epilepsy. Using a cross-sectional observational study design to investigate the spindle deficits in epilepsy and the association between spindle measures and cognitive function may offer a mechanistic biomarker and potential treatment target for cognitive dysfunction in this common neurological disorder.

## 2. Materials and Methods

All the data were from the Epilepsy Center, Lanzhou University Second Hospital. The study was approved by the Ethics Committee of Lanzhou University Second Hospital and conformed to the Declaration of Helsinki principles. Informed consent was obtained from all participants.

### 2.1. Participants

According to the International League Against Epilepsy (ILAE) criteria, right-handed individuals (*n* = 57, age 18-44 years) with clinically diagnosed epilepsy by neurologists were eligible for inclusion in the study. However, this study excluded patients with generalized seizures or secondarily generalized seizures during 12 hours and partial seizures during 6 hours before the evaluation. Patients were excluded if they had the following medical histories: traumatic brain injury, previous cranial surgery, stroke, tumor, neurodegenerative disease, severe mental disorders (e.g., bipolar disorder, schizophrenia, autism, and major depression), and other coexisting severe systemic diseases. Exclusion criteria also included taking medications with a known effect on sleep structure and spindles, such as antipsychotic drugs, central stimulant medications, and sedative-hypnotic drugs, especially benzodiazepines and melatonin receptors agonists, as well as any drug and alcohol abuse/addiction history. Additionally, women who were pregnant, breastfeeding, taking hormonal contraceptives, or receiving hormone replacement therapy were not included in the study. This study also excluded individuals with hearing/vision impairments or BMI ≥ 28 kg/m^2^.

### 2.2. Demographics and Clinical Measure

Demographic data include age, gender, and educational attainment. All participants were right-handed in this study. The total years of formal education were numbered as the years completed after age five. For someone learning full time at conventional speed, repeated years are counted as only one year. And the routine one-year full-time academic load accomplished over several years is also counted as one year.

Clinical features include the onset age of epilepsy, disease duration, seizure type, seizure frequency, the number of antiepileptic drugs (AEDs), history of febrile seizures, and family history of epilepsy. Seizure types are simple partial seizure, complex partial seizure, secondarily generalized seizure, and generalized seizure. Seizure frequency was classified as follows: “at least once a week”; “at least once a month”; “at least once a year”; and “less than once a year.” National Hospital Seizure Severity Scale (NHS3) assesses the seizure severity of each participant. In addition, we measured the height, weight, waist circumference, and hip circumference and then calculated the body mass index (BMI, underweight (<18.5 kg/m^2^), normal weight (18.5-23.9 kg/m^2^), overweight (24-27.9 kg/m^2^), and obese (≥28.0 kg/m^2^)) and waist-to-hip ratio (WHR).

### 2.3. Neuropsychological Assessment

Neuropsychological assessments were conducted in a quiet and comfortable environment on the same day with sleep EEG recording. Global cognitive status was assessed using Montreal Cognitive Assessment (MoCA) and the Mini-Mental State Examination (MMSE).

The MoCA is a brief screening tool to detect mild cognitive impairment (MCI) and has high sensitivity and specificity. We applied the Beijing version of MoCA to evaluate several cognitive domains and then calculated the following cognitive domains index score (CDIS): Memory Index Score (MoCA-MIS, 0-15), Executive Index Score (MoCA-EIS, 0-13), Visuospatial Index Score (MoCA-VIS, 0-7), Language Index Score (MoCA-LIS, 0-6), Attention Index Score (MoCA-AIS, 0-18), and Orientation Index Score (MoCA-OIS, 0-6). For people with less than 12 years of formal education, MoCA total score (MoCA-TS) should be added one point (maximum score of 30).

MMSE is the most commonly used screening tool for dementia, and we employed the Chinese version of MMSE to assess the general cognitive state of participants. The responses of “unable to answer” were considered incorrect answers. MMSE scores range from 0 to 30, with lower scores indicating more severe cognitive symptoms. An unauthorized version of the Chinese MMSE was used by the study team without permission. The MMSE is a copyrighted instrument and may not be used or reproduced in whole or in part, in any form or language, or by any means without written permission of PAR.

According to the recommended cutoffs, cognitive impairment in patients with epilepsy was defined as follows: <23 points (severe cognitive impairment), 23-26 points (mild cognitive impairment), and ≥27 points (no cognitive impairment) for MMSE scores; <20 points (severe cognitive impairment), 20-24 points (mild cognitive impairment), and ≥25 points (no cognitive impairment) for MoCA scores [23, 24].

Furthermore, we also assessed the sleep and mood of participants by employing the Self-Rating Anxiety Scale (SAS), Self-Rating Depression Scale (SDS), and Pittsburgh Sleep Quality Index (PSQI). As for SAS and SDS, higher scores indicate more severe anxiety or depression symptoms, classified as 25-49, no anxiety/depression; 50-59, mild anxiety/depression; 60-69, moderate anxiety/depression; and 70-100, severe anxiety/depression. According to PSQI total score, self-reported sleep quality is classified as 0-5, no sleep disturbance; 6-10, mild sleep disturbance; 11-15, moderate sleep disturbance; and 16-21, severe sleep disturbance. Individuals with severe anxiety, severe depression, or severe sleep disturbance were not included in this study.

### 2.4. EEG Preparation and Acquisition

Participants were instructed not to drink caffeine beverages or strong tea on the day of sleep electroencephalogram (EEG) recording and not to drink alcohol for 24 hours before EEG recording to avoid alcohol- or caffeine-induced changes in EEG. We wiped their scalp with alcohol before placing electrodes to reduce the increase in skin electrical resistance by scalp grease. Sleep EEG recording started approximately within 6 hours after neuropsychological assessment. All participants underwent one-night video EEG monitoring. The purpose, noninvasiveness, and painless nature of examination were fully explained to participants before EEG recording to reduce fear- or nervousness-related emotional responses to minimize the first-night effect.

All scalp electrodes were placed according to the International 10-20 system. EEG data were recorded with a sampling rate of 500 Hz from nineteen scalp electrodes and two reference electrodes (A1 and A2) using Nihon Kohden EEG-1200C and JE-921A amplifier equipment. Two disk electrodes recorded electrooculogram (EOG), one placed 1 cm below the left lateral canthus and another 1 cm above the right lateral canthus. Chin electromyogram (EMG) was applied to detect changes in muscular tension. The recording equipment was identical for all scalp sites and all participants. Impedances were maintained below 10 k*Ω* at all electrodes for most of the night.

The EEG signals were converted to EDF format in Nihon Kohden software, and then, further preprocessed analysis was performed using functions of EEGLAB v2020.0 running on MATLAB R2020b. We excluded the recordings with significant electrocardiogram (ECG) artifacts and seizures or that were contaminated by too many artifacts or disconnected.

### 2.5. Sleep Staging

We employed a deep learning-based sleep staging algorithm U-Sleep to segment any combinations of typical EEG and EOG channels [25]. We process our EEG and EOG raw data set through EEGLAB to obtain the EEG channels used to analyze sleep structure: frontal (F3, F4), central (C3, C4), and occipital (O1, O2), taking A1 and A2 as references, as well as left and right EOG channels. U-Sleep is a fully convolutional neural network that consists of three submodules: encoder module, decoder module, and segment classifier module [25]. The encode module consists of 12 blocks, each consisting of a convolution layer, exponential linear unit, batch normalization, and max pooling. Contrary to the encoder, the decoder module consists of 12 decoder blocks, convolution, nonlinearity, and batch normalization, the stacked feature maps. The segment classifier module aggregates the intermediate representation to a sequence of each sleep stage spanning a fixed 30 seconds. And then, the predictions for each combination of 1 EEG and 1 EOG channel are combined into one final hypnogram. In the experiment, we adopt the pretrained model of U-Sleep to analyze the signals resampled to 128 Hz and output the sleep staging: {W, N1, N2, N3, REM}.

### 2.6. Spindle Detection

Sleep spindles were computationally extracted from each EEG channel with the previously established Spindler algorithm [26]. The detection process mainly includes the following three steps. The single-channel EEG data of the whole night during the N2 sleep stage as the input signal, band-pass filtered (11-17 Hz) by EEGLAB's pop_eegfiltnew and decomposed and reconstructed through matching pursuit with Gabor atoms on a parameter grid. The algorithm then detects sleep spindles in the reconstructed signal and generates spindle property surfaces through the list of spindle events on the parameter grid. Based on the geometry of these surfaces, the algorithm heuristics are applied to select the best parameters. In the experiment, we adopt the default parameter setting of the Spindler algorithm: Gabor atom resolution (Hz), 0.5; scales (2*σ*), [0.125, 0.25, 0.5]; phase (*φ*), 0; atom rate (Ns), [0.01 : 0.01 : 0.4]; threshold (Tb), [0, 10^−5^, 10^−4^, 10^−3^, 10^−2^, 0.02 : 0.01 : 0.1, 0.2 : 0.1 : 1]; frequency range, [11, 17] (sleep); min length, 0.5; min separation, 0.25; max length, 3.0; intersect tolerance, 0.2; onset tolerance, 0.3; and timing tolerance, 0.2 (for more details, please see LaRocco et al. [26]). Sleep spindle detection work was run on MATLAB R2020b in the National Supercomputer Center in Beijing.

The results generated by this algorithm provided sleep spindle number and the timing of the start and end of each spindle. Spindle density of N2 sleep was defined as the number of sleep spindles per minute during NREM sleep stage 2. Another spindle parameter, spindle duration, is calculated as the average duration of each spindle segment. We averaged the values of spindle parameters from the left and right hemispheres and used the mean value for analysis. Correspondence between scalp electrode position and measurement area on the cerebral cortex was confirmed from the previous multisubject study of craniocerebral anatomical correction using the International 10-20 system [27, 28]. The average of the spindle parameter between the left and right hemispheres was named with corresponding craniocerebral anatomical regions, for instance, central (C3, C4), frontal pole (Fp1, Fp2), middle frontal gyrus (F3, F4), pars triangularis of the inferior frontal gyrus (IFGtri) (F7, F8), parietal (P3, P4), occipital (O1, O2), middle temporal (T3, T4), posterior temporal (T5, T6), frontal midline (Fz), central midline (Cz), and parietal midline (Pz).

### 2.7. Statistical Analysis

Data analyses were conducted using IBM SPSS Statistics version 23.0 for Windows. Graphics were generated using SPSS 23.0, GraphPad Prism 9.0, MATLAB R2020b, and Adobe Illustrator 2020 software. Demographics and clinical characteristics of participants were summarized using descriptive statistics.

In spindle parameter comparison between cognitive subgroups, using Levene's test to assess the homogeneity of variances, one-way analysis of variance (ANOVA) was performed when variances were homogeneous, followed by the Tukey-Kramer post hoc tests. In the case of heterogeneous variances, the Welch ANOVA and Games-Howell post hoc test were used for data analysis. Considering we performed multiple ANOVA, *P* values were corrected using the Benjamini–Hochberg method to control the false discovery rate (FDR) at 0.10, and the adjusted *P* value (*P*.adjust) < 0.10 was considered statistically significant [29, 30].

Multiple linear regressions were applied to analyze the associations between the *Z*-scores of spindle characteristics in each brain region and the *Z*-scores of cognitive outcomes. Raw data of cognitive scores and spindle characteristics (density and duration) were normalized by conversion to *Z*-scores using the means and standard deviations (SD). Gender and age were included in the regression model as obligatory confounders for statistically controlling. Multivariate models were also adjusted for possible confounders through initial analysis of potential covariates. Correlation analyses, independent-samples *t*-test, and one-way ANOVA (for homoscedasticity) or Welch ANOVA (for heteroscedasticity) were employed to screen potential covariates of the multiple regression model. Categorical variables were coded as dummy variables. Variance inflation factor (VIF) was used to confirm absence of multicollinearity between these variables (VIF < 2). Statistical significance was set at *P* < 0.05; statistical trends were set at *P* < 0.10. Since we performed a large number of regressions between cognitive function and topographical spindle characteristics, the Benjamini and Hochberg method was used to correct the *P* values, and *P*.adjust < 0.10 was considered still statistically significant after correction [29, 30].

## 3. Results

### 3.1. Participant Characteristics

A total of 57 adults with epilepsy were included in the study, with 57.9% females and an average age of 27.18 years. According to the MMSE score, 36 participants (63.2%) had no cognitive impairment, 12 participants (21.1%) had mild cognitive impairment, and 9 participants (15.8%) had severe cognitive impairment. According to the MoCA score, 24 participants (42.1%) had no cognitive impairment, 20 participants (35.1%) had mild cognitive impairment, and 13 participants (22.8%) had severe cognitive impairment. The demographic and clinical characteristics and sleep architecture variables of participants are provided in Table 1.

### 3.2. Spindle Characteristics between Cognitive Subgroups

**Figure 1:**
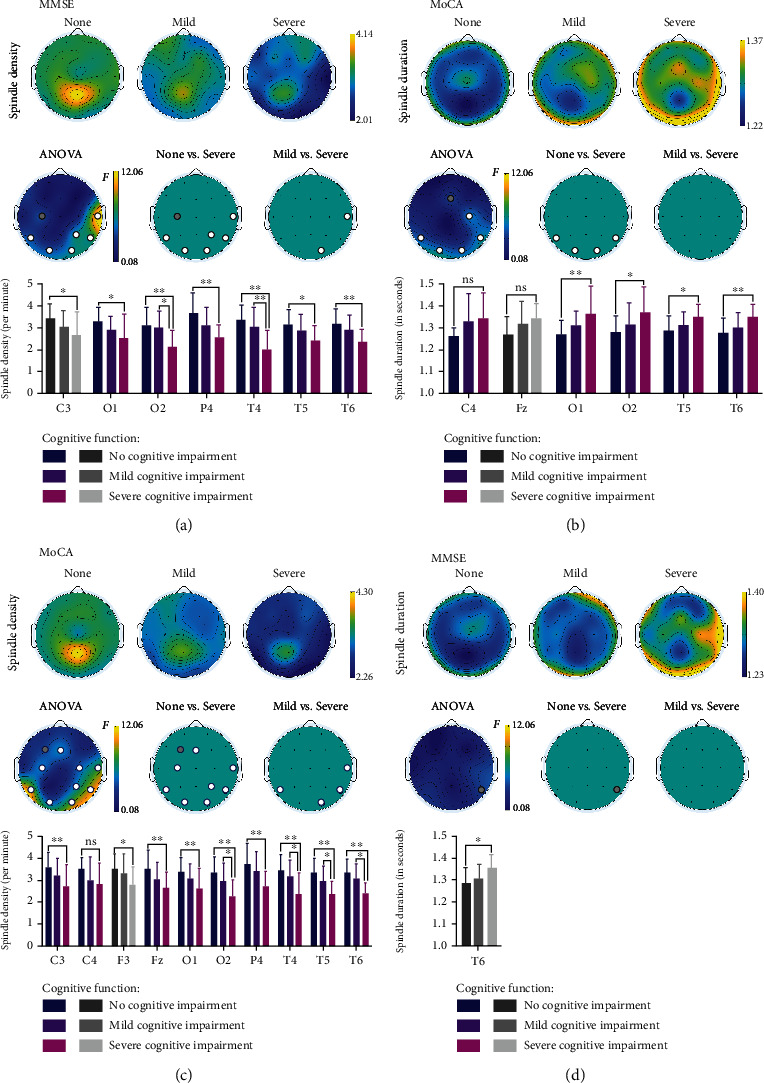
Spindle characteristics during N2 sleep. First row of each graphic: the topographic distribution of average spindle parameters at scalp level: (a) spindle density and (d) spindle duration of cognitive impairment grouping by MMSE, including none (≥27 points, *n* = 36), mild (23-26 points, *n* = 12), and severe (<23 points, *n* = 9); (c) spindle density and (b) spindle duration of cognitive impairment grouping by MoCA, including none (≥25 points, *n* = 24), mild (20-24 points, *n* = 20), and severe (<20 points, *n* = 13). The maps are based on 19 derivations of the International 10-20 system (electrodes positions indicated by black dots). Second row of each graphic: statistical maps (*F*-values) of ANOVA (none vs. mild vs. severe) about spindle parameter. For the derivations exhibiting significant between-group differences employing ANOVA (*P* < 0.05), white dots indicate statistically significant after Benjamini and Hochberg correction (*P*.adjust < 0.10), and grey dots indicate no statistical significance after correction (*P*.adjust ≥ 0.10). The significant difference in the post hoc test (none vs. severe, mild vs. severe) is also indicated by white/grey dots correspondingly (*P* < 0.05). Third row of each graphic: histograms (mean ± standard deviation) of the spindle parameter in each derivation show the significant differences among cognitive subgroups in ANOVA. Colored histograms indicate still statistical significance after Benjamini and Hochberg correction (*P*.adjust < 0.10), and the gray histograms with no statistical significance after correction (*P*.adjust ≥ 0.10). Statistical significance of the post hoc test is indicated with the following: ^∗^*P* < 0.05, ^∗∗^*P* < 0.01, and ns: no statistical significance.

Figure 1 shows the topographic distribution of spindle characteristics during N2 sleep. ANOVA and the post hoc test revealed significant differences among three cognitive subgroups in spindle parameters. There were no significant differences among cognitive subgroups in participant characteristics and sleep variables (see Tables S1 and S2 in the Supplementary Material for comprehensive data analysis).

For MMSE cognition grouping, the significant differences on spindle density among cognitive subgroups were distributed in C3 (one-way ANOVA, *F* = 3.785, *P* = 0.029), O1 (one-way ANOVA, *F* = 4.240, *P* = 0.019), O2 (one-way ANOVA, *F* = 5.781, *P* = 0.005), P4 (one-way ANOVA, *F* = 6.287, *P* = 0.004), T4 (one-way ANOVA, *F* = 12.0646, *P* < 0.001), T5 (one-way ANOVA, *F* = 4.129, *P* = 0.021), and T6 (one-way ANOVA, *F* = 6.182, *P* = 0.004) (Figure 1(a)). Except for C3 (*P*.adjust = 0.105), these differences in spindle density between subgroups remained statistically significant after Benjamini and Hochberg correction (*P*.adjust < 0.10): O1, *P*.adjust = 0.090; O2, *P*.adjust = 0.048; P4, *P*.adjust = 0.051; T4, *P*.adjust = 0.002; T5, *P*.adjust = 0.089; and T6, *P*.adjust = 0.043 (Figure 1(a)). The post hoc tests showed that the spindle density of participants with severe cognitive impairment for MMSE score was lower than both no cognitive impairment (C3, *P* = 0.031; O1, *P* = 0.020; O2, *P* = 0.004; P4, *P* = 0.004; T4, *P* < 0.001; T5, *P* = 0.019; and T6, *P* = 0.003) and mild cognitive impairment (O2, *P* = 0.035; T4, *P* = 0.007) (Figure 1(a)). However, there was no significant difference in spindle density between the no cognitive impairment group and mild cognitive impairment group for MMSE scores (*P* > 0.05).

The significant differences in spindle density between cognitive subgroups for MoCA scores were distributed in C3 (one-way ANOVA, *F* = 5.106, *P* = 0.009), C4 (Welch ANOVA, *F* = 4.426, *P* = 0.023), F3 (one-way ANOVA, *F* = 3.651, *P* = 0.033), Fz (one-way ANOVA, *F* = 5.418, *P* = 0.007), O1 (one-way ANOVA, *F* = 4.760, *P* = 0.012), O2 (one-way ANOVA, *F* = 8.368, *P* = 0.001), P4 (one-way ANOVA, *F* = 5.605, *P* = 0.006), T4 (one-way ANOVA, *F* = 7.553, *P* = 0.001), T5 (one-way ANOVA, *F* = 10.095, *P* < 0.001), and T6 (one-way ANOVA, *F* = 9.625, *P* < 0.001) (Figure 1(c)). These results were still statistically significant after Benjamini-Hochberg correction (C3, *P*.adjust = 0.053; C4, *P*.adjust = 0.087; Fz, *P*.adjust = 0.053; O1, *P*.adjust = 0.065; O2, *P*.adjust = 0.019; P4, *P*.adjust = 0.051; T4, *P*.adjust = 0.015; T5, *P*.adjust = 0.004; and T6, *P*.adjust = 0.004), excluding F3 (*P*.adjust = 0.114) (Figure 1(c)). In addition, the post hoc tests indicated that the spindle density in the severe cognitive impairment subgroup for MoCA scores was lower than that in both the no cognitive impairment subgroup (C3, *P* = 0.007; F3, *P* = 0.025; Fz, *P* = 0.006; O1, *P* = 0.009; O2, *P* < 0.001; P4, *P* = 0.004; T4, *P* = 0.001; T5, *P* < 0.001; and T6, *P* < 0.001) and mild cognitive impairment subgroup (O2, *P* = 0.040; T4, *P* = 0.018; T5, *P* = 0.032; and T6, *P* = 0.011) (Figure 1(c)). The severe cognitive impairment subgroup showed no significant difference in C4 spindle density (*P* = 0.058) compared with the no cognitive impairment subgroup for MoCA scores. Moreover, there was no significant difference in spindle density between the no cognitive impairment subgroup and the mild cognitive impairment subgroup (*P* > 0.05).

The significant difference in spindle duration between cognitive subgroups for MMSE scores was distributed in T6 (one-way ANOVA, *F* = 3.473, *P* = 0.038), and the post hoc test showed that T6 spindle duration in the severe cognitive impairment subgroup was more than that in the no cognitive impairment subgroup (*P* = 0.030), but no longer significant after Benjamini-Hochberg correction (*P*.adjust = 0.126) (Figure 1(d)). There was no significant difference between the severe cognitive impairment subgroup and no cognitive impairment subgroup in C4 (*P* = 0.063), T4 (*P* = 0.053), and T5 (*P* = 0.080) spindle duration. The post hoc test indicated no significant difference in spindle duration between the mild cognitive impairment subgroup and the no/severe cognitive impairment subgroup for MMSE scores (*P* > 0.05).

The significant differences in spindle duration between cognitive subgroups for MoCA scores were distributed in C4 (Welch ANOVA, *F* = 4.970, *P* = 0.017), Fz (one-way ANOVA, *F* = 3.373, *P* = 0.042), O1 (one-way ANOVA, *F* = 5.328, *P* = 0.008), O2 (one-way ANOVA, *F* = 4.199, *P* = 0.020), T5 (one-way ANOVA, *F* = 4.107, *P* = 0.022), and T6 (one-way ANOVA, *F* = 5.465, *P* = 0.007) (Figure 1(c)). In addition to Fz (*P*.adjust = 0.133), these differences in spindle duration between subgroups were still statistically significant after *P* values correction (C4, *P*.adjust = 0.086; O1, *P*.adjust = 0.051; O2, *P*.adjust = 0.089; T5, *P*.adjust = 0.088; and T6, *P*.adjust = 0.048). Furthermore, the results of the post hoc test indicated that spindle duration in the severe cognitive impairment subgroup was more than that in the no cognitive impairment subgroup for MoCA scores (O1, *P* = 0.006; O2, *P* = 0.015; T5, *P* = 0.017; and T6, *P* = 0.005), and there was no significant difference between the severe cognitive impairment subgroup and no cognitive impairment subgroup in C4 (*P* = 0.076) and Fz (*P* = 0.050) spindle duration (Figure 1(c)). The post hoc test indicated no significant difference in spindle duration between the mild cognitive impairment subgroup and the no/severe cognitive impairment subgroup for MoCA scores (*P* > 0.05).

### 3.3. Initial Analyses between Cognitive Outcomes and Potential Covariates

As Table 2 shows, education years were moderately to highly correlated with each cognitive score (*r*, 0.414-0.710; *P* < 0.05). Epilepsy duration only correlated moderately with the MMSE total score (*r* = −0.331, *P* = 0.012), excluding other cognitive performance (all *P* > 0.05 via correlation analysis). In addition, there were no significant correlations between each cognitive score and age of onset, the number of AEDs, NHS3 score, and WHR in our study sample (all *P* > 0.05 via correlation analysis).

**Figure 2:**
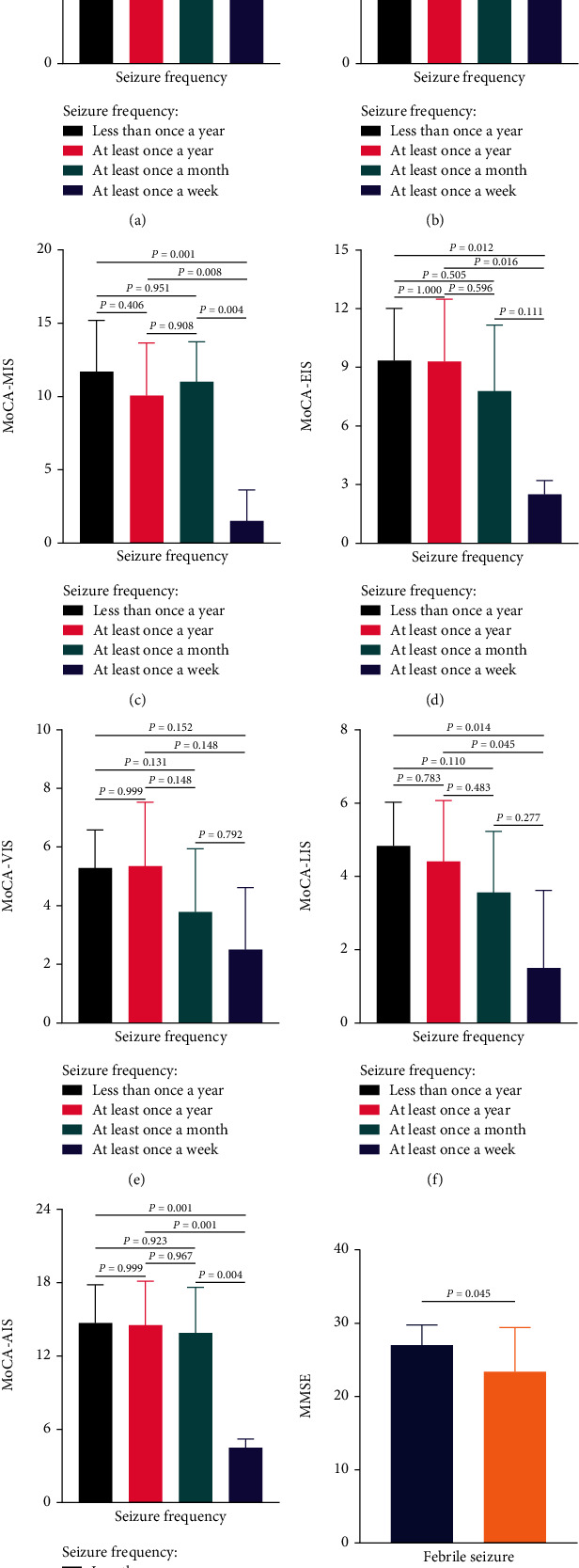
Subgroup differences among seizure frequency and history of febrile seizure in cognitive outcomes. (a–g) The results of the post hoc test among seizure frequency subgroups in each cognitive score. MMSE: Mini-Mental State Examination total score; MoCA-TS: Montreal Cognitive Assessment total score; MoCA-MIS: Memory Index Score; MoCA-EIS: Executive Index Score; MoCA-VIS: Visuospatial Index Score; MoCA-LIS: Language Index Score; MoCA-AIS: Attention Index Score. (h) MMSE total score of participants who have a history of febrile seizure is lower than that of the absence of febrile seizure via independent-samples *t*-test.

The differences between seizure frequency subgroups in cognitive scores were statistically significant via one-way ANOVA, except for MoCA-OIS (*F* = 0.238, *P* = 0.869). Specifically, MMSE (*F* = 5.162, *P* = 0.003), MoCA-TS (*F* = 3.951, *P* = 0.013), MoCA-MIS (*F* = 5.894, *P* = 0.002), MoCA-EIS (*F* = 3.929, *P* = 0.013), MoCA-VIS (*F* = 3.219, *P* = 0.030), MoCA-LIS (*F* = 4.542, *P* = 0.007), and MoCA-AIS (*F* = 5.863, *P* = 0.002). Figures 2(a)–2(g) show the results of the post hoc test among seizure frequency subgroups in each cognitive score. MMSE total score of participants with a history of febrile seizure was significantly lower than that of the absence of febrile seizure via independent-samples *t*-test (23.36 ± 6.03 vs. 27.00 ± 2.78, *t* = 2.185, *P* = 0.045) (Figure 2(h)). The differences between febrile seizure subgroups in other cognitive scores failed to meet statistical significance (all *P* > 0.05 via independent-samples *t*-test).

### 3.4. Association between Cognitive Function and N2 Sleep Spindle Characteristic

**Figure 3:**
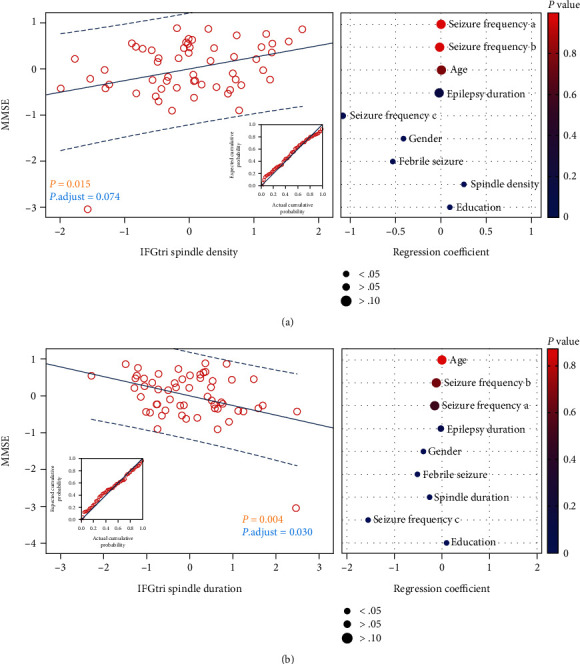
Multiple linear regression with the *Z*-score of MMSE as the dependent variable and the *Z*-score of spindle parameter as the independent variable. Blue solid (dashed) lines indicate model fits (95% confidence intervals) of the partial regression plot. *P* is the uncorrected *P* value of the spindle parameter, and *P*.adjust is the corrected *P* value via the Benjamini-Hochberg method of spindle parameter. Sleep spindle characteristics with significant *P*-values (<0.05) and *P*.adjust (<0.10) are highlighted in yellow and blue, respectively. IFGtri: pars triangularis of inferior frontal gyrus. Employing dummy variables for categorical variables, gender takes the male as a reference, history of febrile seizure with absence as a reference, and seizure frequency with less than once a year as a reference. Seizure frequencies a, b, and c represent at least once a year, at least once a month, and at least once a week.

MMSE was positively associated with spindle density in IFGtri (*β* (95%CI) = 0.253 (0.051~0.455), *P* = 0.015) (Figure 3(a)) and negatively associated with spindle duration in IFGtri (*β* (95%CI) = −0.262 (-0.435~-0.088), *P* = 0.004) (Figure 3(b)). These associations remained statistically significant (IFGtri spindle density, *P*.adjust = 0.074; IFGtri spindle duration, *P*.adjust = 0.030), after correcting *P* value applying the Benjamini and Hochberg method.

**Figure 4:**
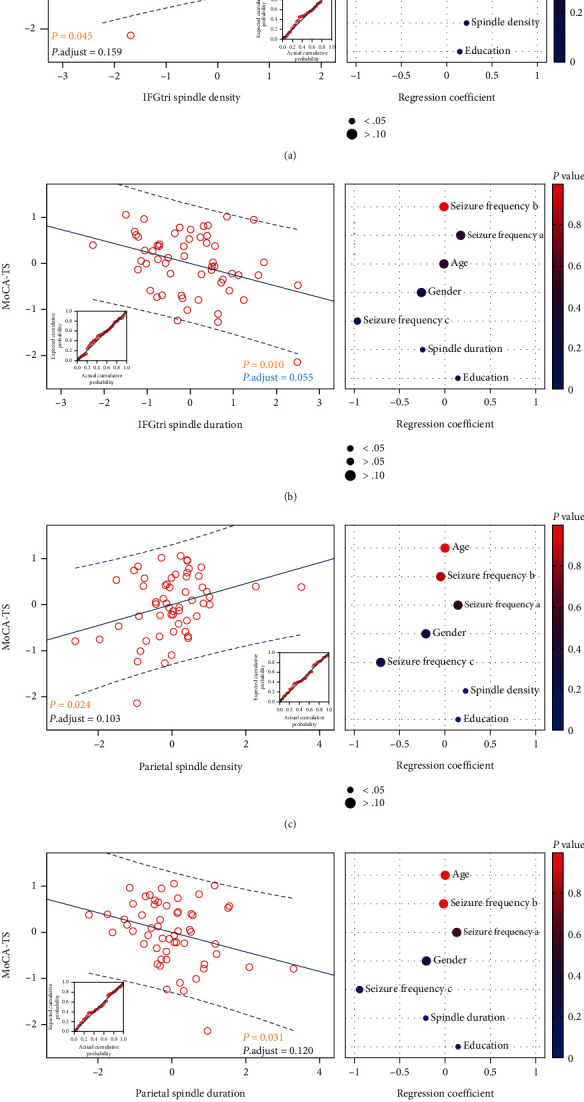
Multiple linear regression with the *Z*-score of MoCA-TS as the dependent variable and the *Z*-score of spindle parameter as the independent variable. Blue solid (dashed) lines indicate model fits (95% confidence intervals) of the partial regression plot. *P* is the uncorrected *P* value of the spindle parameter, and *P*.adjust is the corrected *P* value via the Benjamini-Hochberg method of spindle parameter. Sleep spindle characteristics with significant *P*-values (<0.05) and *P*.adjust (<0.10) are highlighted in yellow and blue, respectively. IFGtri: pars triangularis of inferior frontal gyrus. Employing dummy variables for categorical variables, gender takes the male as a reference and seizure frequency with less than once a year as a reference. Seizure frequencies a, b, and c represent at least once a year, at least once a month, and at least once a week.

MoCA-TS was positively associated with spindle density in IFGtri (*β* (95%CI) = 0.217 (0.005~0.430), *P* = 0.045, and *P*.adjust = 0.159) (Figure 4(a)), negatively associated with spindle duration in IFGtri (*β* (95%CI) = −0.246 (-0.430~-0.062), *P* = 0.010, and *P*.adjust = 0.055) (Figure 4(b)), positively associated with spindle density in parietal (*β* (95%CI) = 0.227 (0.031~0.424), *P* = 0.024, and *P*.adjust = 0.103) (Figure 4(c)), and negatively associated with spindle duration in parietal (*β* (95%CI) = −0.213 (-0.405~-0.020), *P* = 0.031, and *P*.adjust = 0.120) (Figure 4(d)). After *P* value correction, only the negative association between spindle duration in IFGtri and MoCA-TS was still statistically significant (*P*.adjust = 0.055).

**Figure 5:**
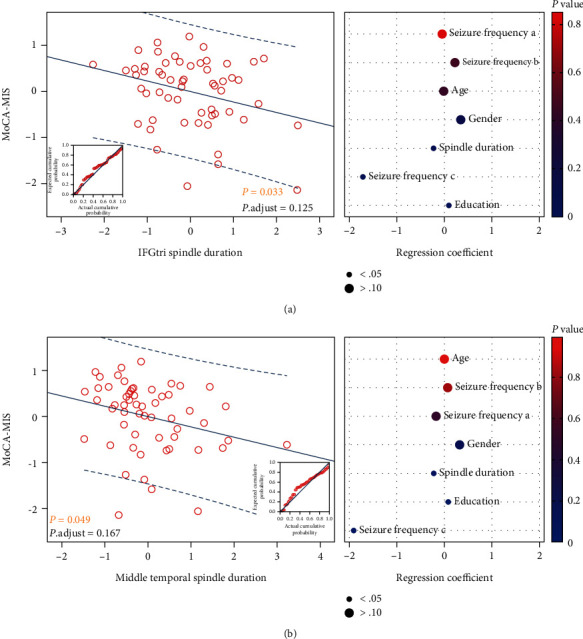
Multiple linear regression with the *Z*-score of MoCA-MIS as the dependent variable and the *Z*-score of spindle parameter as the independent variable. Blue solid (dashed) lines indicate model fits (95% confidence intervals) of the partial regression plot. *P* is the uncorrected *P* value of the spindle parameter, and *P*.adjust is the corrected *P* value via the Benjamini-Hochberg method of spindle parameter. Sleep spindle characteristics with significant *P*-values (<0.05) and *P*.adjust (<0.10) are highlighted in yellow and blue, respectively. IFGtri: pars triangularis of inferior frontal gyrus. Employing dummy variables for categorical variables, gender takes the male as a reference and seizure frequency with less than once a year as a reference. Seizure frequencies a, b, and c represent at least once a year, at least once a month, and at least once a week.

For multiple linear regression, MoCA-MIS was negatively associated with spindle duration in IFGtri (*β* (95%CI) = −0.228 (-0.437~-0.019), *P* = 0.033, and *P*.adjust = 0.125) (Figure 5(a)) and negatively associated with spindle duration in middle temporal (*β* (95%CI) = −0.224 (-0.447~-0.001), *P* = 0.049, and *P*.adjust = 0.167) (Figure 5(b)). However, none were statistically significant, after correcting *P* value by the Benjamini and Hochberg method (all *P*.adjust > 0.10).

**Figure 6:**
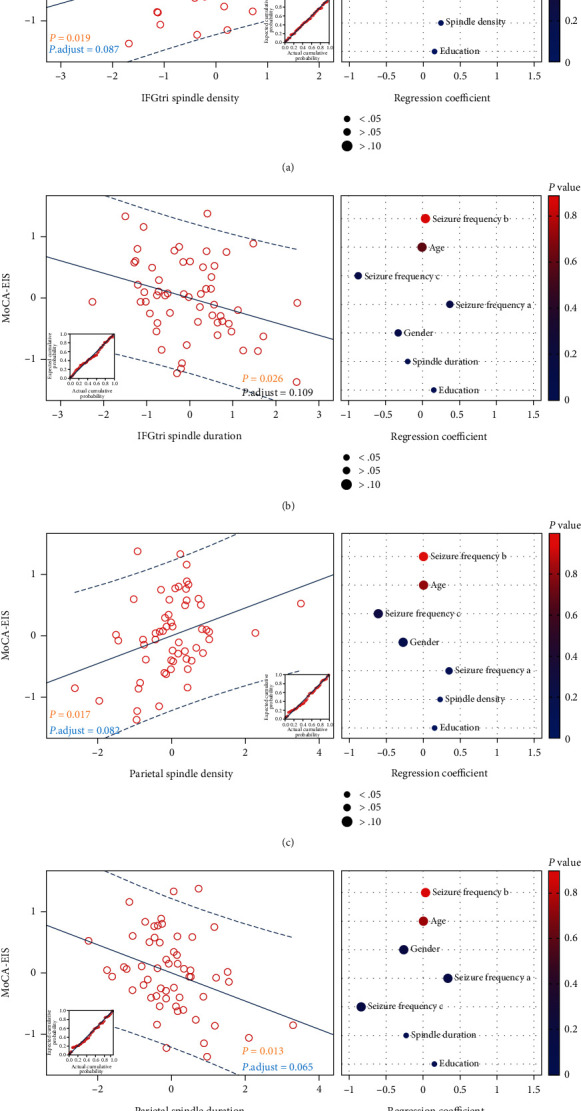
Multiple linear regression with the *Z*-score of MoCA-EIS as the dependent variable and the *Z*-score of spindle parameter as the independent variable. Blue solid (dashed) lines indicate model fits (95% confidence intervals) of the partial regression plot. *P* is the uncorrected *P* value of the spindle parameter, and *P*.adjust is the corrected *P* value via the Benjamini-Hochberg method of spindle parameter. Sleep spindle characteristics with significant *P*-values (<0.05) and *P*.adjust (<0.10) are highlighted in yellow and blue, respectively. IFGtri: pars triangularis of inferior frontal gyrus. Employing dummy variables for categorical variables, gender takes the male as a reference and seizure frequency with less than once a year as a reference. Seizure frequencies a, b, and c represent at least once a year, at least once a month, and at least once a week.

MoCA-EIS was positively associated with spindle density in IFGtri (*β* (95%CI) = 0.238 (0.041~0.435), *P* = 0.019, and *P*.adjust = 0.087) (Figure 6(a)), negatively associated with spindle duration in IFGtri (*β* (95%CI) = −0.202 (-0.379~-0.025), *P* = 0.026, and *P*.adjust = 0.109) (Figure 6(b)), positively associated with spindle density in parietal (*β* (95%CI) = 0.227 (0.042~0.411), *P* = 0.017, and *P*.adjust = 0.082) (Figure 6(c)), and negatively associated with spindle duration in parietal (*β* (95%CI) = −0.230 (-0.409~-0.051), *P* = 0.013, and *P*.adjust = 0.065) (Figure 6(d)). After *P* value correction, these findings remained statistically significant (*P*.adjust < 0.10), except for spindle duration in IFGtri (*P*.adjust = 0.109).

**Figure 7:**
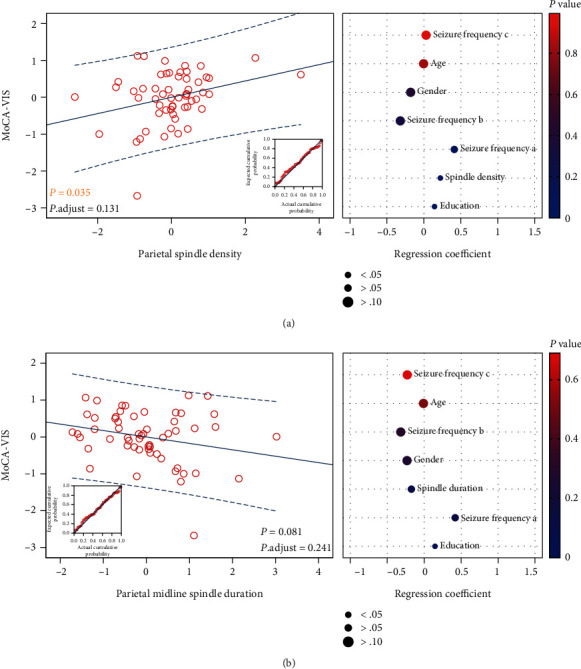
Multiple linear regression with the *Z*-score of MoCA-VIS as the dependent variable and the *Z*-score of spindle parameter as the independent variable. Blue solid (dashed) lines indicate model fits (95% confidence intervals) of the partial regression plot. *P* is the uncorrected *P* value of the spindle parameter, and *P*.adjust is the corrected *P* value via the Benjamini-Hochberg method of spindle parameter. Sleep spindle characteristics with significant *P*-values (<0.05) and *P*.adjust (<0.10) are highlighted in yellow and blue, respectively. Employing dummy variables for categorical variables, gender takes the male as a reference and seizure frequency with less than once a year as a reference. Seizure frequencies a, b, and c represent at least once a year, at least once a month, and at least once a week.

For multiple linear regression, MoCA-VIS was positively associated with spindle density in parietal (*β* (95%CI) = 0.222 (0.016~0.427), *P* = 0.035, and *P*.adjust = 0.131) (Figure 7(a)), showed a statistically negative trend with spindle duration in the parietal midline (*β* (95%CI) = −0.174 (-0.371~0.022), *P* = 0.081, and *P*.adjust = 0.241) (Figure 7(b)). However, these results failed to reach statistically significance, after correcting *P* value by the Benjamini and Hochberg method (all *P*.adjust > 0.10).

**Figure 8:**
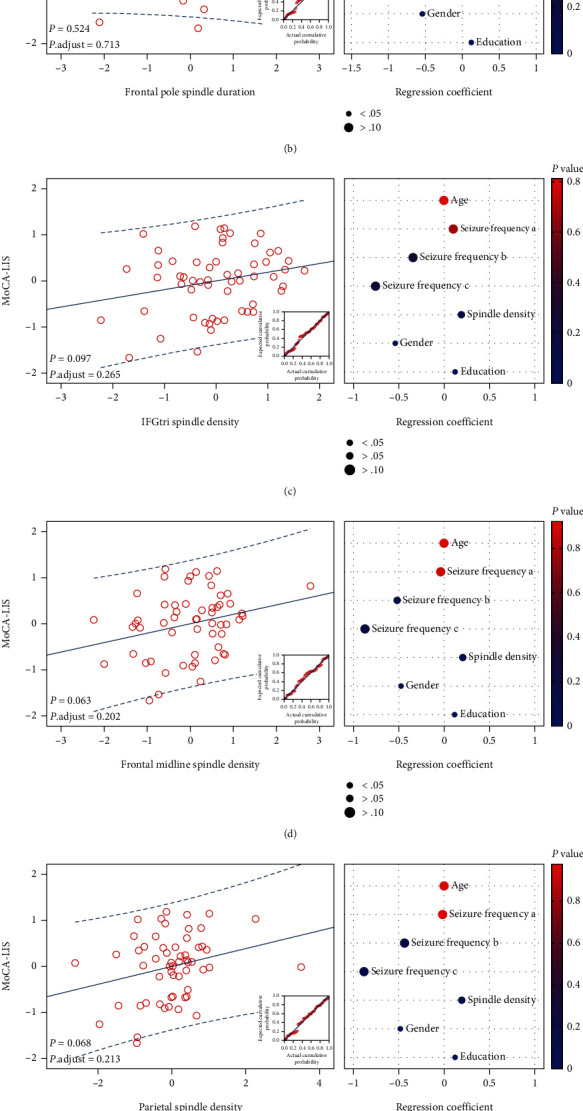
Multiple linear regression with the *Z*-score of MoCA-LIS as the dependent variable and the *Z*-score of spindle parameter as the independent variable. Blue solid (dashed) lines indicate model fits (95% confidence intervals) of the partial regression plot. *P* is the uncorrected *P* value of the spindle parameter, and *P*.adjust is the corrected *P* value via the Benjamini-Hochberg method of spindle parameter. Sleep spindle characteristics with significant *P*-values (<0.05) and *P*.adjust (<0.10) are highlighted in yellow and blue, respectively. IFGtri: pars triangularis of inferior frontal gyrus. Employing dummy variables for categorical variables, gender takes the male as a reference and seizure frequency with less than once a year as a reference. Seizure frequencies a, b, and c represent at least once a year, at least once a month, and at least once a week.

In our multiple regression models, MoCA-LIS was positively associated with spindle density in the frontal pole (*β* (95%CI) = 0.226 (0.027~0.425), *P* = 0.027, and *P*.adjust = 0.111) (Figure 8(a)) but had no significant association with spindle duration in the frontal pole (*β* (95%CI) = −0.068 (-0.281~0.145), *P* = 0.524, and *P*.adjust = 0.713) (Figure 8(b)). MoCA-LIS showed a statistically positive trend with spindle density in IFGtri (*β* (95%CI) = 0.189 (-0.035~0.413), *P* = 0.097, and *P*.adjust = 0.265) (Figure 8(c)), spindle density in frontal midline (*β* (95%CI) = 0.205 (-0.011~0.420), *P* = 0.063, and *P*.adjust = 0.202) (Figure 8(d)), and spindle density in parietal (*β* (95%CI) = 0.194 (-0.015~0.402), *P* = 0.068, and *P*.adjust = 0.213) (Figure 8(e)). MoCA-LIS also showed a statistically negative trend with spindle duration in the parietal midline (*β* (95%CI) = −0.170 (-0.367~0.028), *P* = 0.091, and *P*.adjust = 0.255) (Figure 8(f)). Notably, all these findings on MoCA-LIS did not meet statistical significance after *P* value correction by the Benjamini and Hochberg method (all *P*.adjust > 0.10).

**Figure 9:**
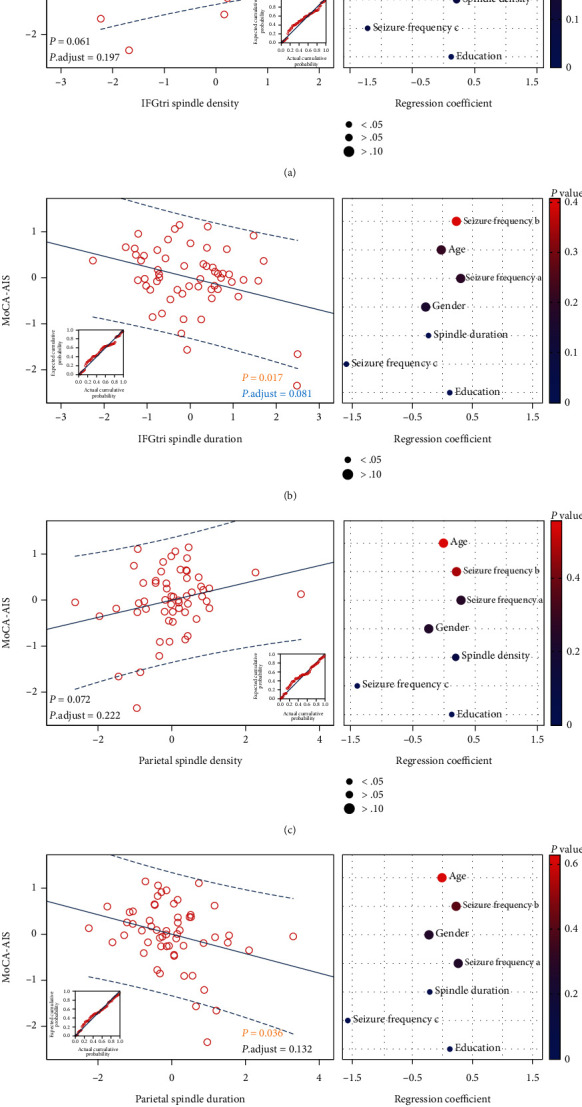
Multiple linear regression with the *Z*-score of MoCA-AIS as the dependent variable and the *Z*-score of spindle parameter as the independent variable. Blue solid (dashed) lines indicate model fits (95% confidence intervals) of the partial regression plot. *P* is the uncorrected *P* value of the spindle parameter, and *P*.adjust is the corrected *P* value via the Benjamini-Hochberg method of spindle parameter. Sleep spindle characteristics with significant *P*-values (<0.05) and *P*.adjust (<0.10) are highlighted in yellow and blue, respectively. IFGtri: pars triangularis of inferior frontal gyrus. Employing dummy variables for categorical variables, gender takes the male as a reference and seizure frequency with less than once a year as a reference. Seizure frequencies a, b, and c represent at least once a year, at least once a month, and at least once a week.

MoCA-AIS was negatively associated with spindle duration in IFGtri (*β* (95%CI) = −0.233 (-0.423~-0.043), *P* = 0.017, and *P*.adjust = 0.081) (Figure 9(b)) and also negatively associated with spindle duration in parietal (*β* (95%CI) = −0.212 (-0.409~-0.014), *P* = 0.036, and *P*.adjust = 0.132) (Figure 9(d)). MoCA-AIS showed a statistically positive trend with spindle density in IFGtri (*β* (95%CI) = 0.208 (-0.010~0.427), *P* = 0.061, and *P*.adjust = 0.197) (Figure 9(a)) and spindle density in parietal (*β* (95%CI) = 0.187 (-0.018~0.393), *P* = 0.072, and *P*.adjust = 0.222) (Figure 9(c)). After *P* value correction by the Benjamini and Hochberg method, only the negative association between spindle duration in IFGtri and MoCA-AIS remained statistically significant (*P*.adjust = 0.081) (Figure 9(b)). Furthermore, there were no significant associations between MoCA-OIS and sleep spindle characteristics in our regression models. The scores of MoCA-OIS mainly focused on 5-6 in our study sample, which may reflect the limitations of our methods. The rest results of multiple linear regression were detailed in the supplementary materials (see Tables S3–S9 in the Supplementary Material for comprehensive data analysis).

## 4. Discussion

Epilepsy is a common brain disorder, affecting approximately 70 million people worldwide [31]. Two-thirds of seizures might be controlled by antiepileptic drugs, whereas the medications do not improve the long-term prognosis of people with epilepsy. Comorbidities are considered significant in both etiology and prognostic markers [32]. Growing evidence suggests an association between cognitive impairment and epilepsy. Previous studies mainly focused on seizures, interictal epileptiform discharges, treatment-related factors, and psychosocial factors. The pathophysiological mechanism of cognition and sleep microstructure is less elucidated in people with epilepsy. This study explored the association between sleep spindles and epilepsy-related cognitive impairment from a neuropsychological perspective. We found that epilepsy with severe cognitive impairment had lower spindle density and relatively long spindle duration compared with no cognitive impairment and mild cognitive impairment. In our multiple linear regression models, the global cognitive status is related to spindle characteristics in adult epilepsy. After *P* value correction by the Benjamini and Hochberg method, MoCA-EIS and MoCA-AIS also have a relationship with the sleep spindle in IFGtri or parietal at the scalp level.

Sleep spindles are characteristic EEG signatures of N2 sleep. Animal studies have shown that the neuronal substrates of spindles involve a cortex-TRN-thalamus circuit [13]. Specifically, it is well established that the TRN is the spindle pacemaker [13]. TRN is mainly composed of a relatively thin layer of neurons, and the major cell type is GABAergic neurons [33]. As a derivative of the ventral thalamus, TRN wraps the lateral and anterior dorsal thalamus and, to some extent, the ventral surface of the dorsal thalamus [34]. Due to its unique anatomical position, TRN receives excitatory afferents from cortical and thalamic neurons and releases inhibitory neurotransmitter *γ*-aminobutyric acid to all thalamic nuclei [35], forming a feedback loop mainly regulated by GABAergic and glutamatergic neurotransmitters [36]. Sleep spindles are amplified and spread through the circuits between corticocortical and thalamocortical.

Here, we showed the significant differences in spindle characteristics at the scalp level among cognitive subgroups in adult epilepsy (Figure 1). We found that epilepsy with severe cognitive impairment had lower spindle density and relatively long spindle duration compared with no cognitive impairment and mild cognitive impairment. Altered spindles suggest thalamic cortex or TRN dysfunctions in adults with epilepsy. These results are consistent with the growing body of evidence that supports the dysfunction of thalamocortical circuits in epilepsy [4, 10]. Subtle structural abnormalities in both the thalamus and thalamocortical white matter are observed in patients with epilepsy [37, 38]. Previous studies have shown that sleep spindles are ubiquitous in the cortex, and spatiotemporal features are affected by the underlying cortex [20–22]. The difference between cognitive subgroups in spindle density was relatively distributed wide, mainly involving central, occipital, parietal, middle temporal, and posterior temporal, compared to the difference in spindle duration mainly distributed in occipital and posterior temporal. While preliminary, our observations and comparisons suggest that spindle density could be more sensitive to epilepsy-related cognitive impairment than duration, and spindle plasticity provides possibilities for targeted intervention. Compared with no/mild cognitive impairment, sleep spindle activity was impaired substantially in epilepsy patients who developed severe cognitive impairment. The reasons for this change in spindle parameters as cognitive dysfunction progresses are unknown, and we can only speculate as to the potential causes. Spindle deficits in epilepsy may result from impairments in either TRN/thalamus circuits or corticothalamic afferents to these intrathalamic circuits. For spindle parameters, the number/spindle density, which likely reflects the pacemaker activity of TRN/thalamus circuits, was reduced in epilepsy with severe cognitive impairment compared to no/mild cognitive impairment. Our results on spindles are also in line with a previous study showing the decrease of sleep spindle density in Alzheimer's disease and MCI [39]. However, we did not find a significant difference in spindle parameters between mild cognitive impairment and no cognitive impairment in adults with epilepsy. We speculate that the spindle parameters of epilepsy patients may not have changed very significantly at the early stage of clinical cognitive decline, or our analytical methods may not be sensitive enough to reveal subtle changes on the sleep spindle.

This study also explored the association between spindle characteristics during N2 sleep and cognitive functioning in adults with epilepsy, considering confounding factors including age, gender, education years, and seizure frequency. For instance, in our multiple regression models, the total score of MMSE (Figures 3(a) and 3(b)) and MoCA-TS (Figure 4(b)) remained to have statistically significant associations with spindle characteristics in IFGtri, after the *P* value correction via the Benjamini and Hochberg method. In other words, this study suggested that global cognitive status was associated with N2 sleep spindle characteristics (both density and duration) in the cognitive assessment of adult epilepsy. Our observations are broadly consistent with a previous study among middle-aged and elderly adults with sedentary habits showing the positive association between NREM stage 2 spindle density in central and frontal regions and MoCA score [40]. Furthermore, a previous study showed a positive correlation between parietal spindle density and MMSE score in Alzheimer's disease and MCI [39], while the relationship between parietal spindle parameters and global cognitive status (Figures 4(c) and 4(d)) failed to reach statistical significance after correcting *P* value. Besides that, in our multiple regression models, sleep spindle density showed a tendency towards a positive correlation with a few cognitive scores, which is generally consistent with prior studies [39, 40]. The present study also suggested a trending negative correlation between several cognitive scores and spindle duration. We also found that adults with epilepsy and severe cognitive impairment had relatively long spindle duration compared to mild cognitive impairment and normal cognition. In line with previous studies, extreme spindles have been reported in people with mental retardation, and this abnormal EEG waveform has higher amplitudes and longer durations than in normal individuals [41–43]. Previous studies have shown the mechanisms of corticothalamic feedback controlling spindle duration in vivo [44]. Corticothalamic feedback mediates spindle termination with weak calcium upregulation, has a relative impact on thalamic reticular neurons and thalamocortical relay neurons, and contributes to spindle termination by desynchronizing of thalamic neurons [44]. Given the limited research available, we can only speculate about the potential mechanisms mediating long spindle duration in epilepsy patients with cognitive impairment, which may relate to thalamocortical dysfunction. Future studies will be needed to examine brain connectivity, cognitive processes, and sleep spindles to further elucidate the functional significance of these observed associations.

In addition, we were trying to understand the potential influence of anatomical regions with diverse functions on the sleep spindle in terms of MoCA-CDIS. Our results suggest that MoCA-EIS was associated with spindle duration in parietal and spindle density in IFGtri and parietal (Figure 6). Sleep spindles are an event that occurs throughout the brain, while the spatiotemporal characteristics are not uniform, depending on cortical location [22]. Executive functions (also called cognitive control or executive control) are a series of top-down mental processes required when you have to concentrate, which would be ill-advised, inadequate, or impossible when going on automatically or depending on instinct or intuition [45]. There are three core executive functions widely recognized: inhibitory control (including self-control, selective attention, and cognitive inhibition), working memory, and cognitive flexibility, which require the participation of the prefrontal cortex and are necessary for goal-oriented behavior. Complex or higher-order executive functions are constructed based on these, such as reasoning, planning, and problem-solving [45]. The task of Tower of Hanoi mainly involves executive functions such as problem-solving and planning. A previous study about event-related functional magnetic resonance imaging in healthy adults has shown the activation confined to a frontoparietal system during Tower of Hanoi task performance, and the activation involves two distinct time courses [46]. Specifically, one activation varies with the target processing operation parameters, and areas showing parametric relationships involve the frontoparietal system. Another activation became apparent only during goal-intensive processing trials, and the left inferior frontal gyrus is preferentially involved in this activation [46]. Consistent with these findings, our results also suggested the relationship between MoCA-EIS and spindle parameters in IFGtri and parietal regions. Petrides and Pandya have reported rich interconnections between the parietal and frontal areas [47]. Many neuroimaging studies have implicated the coactivation of prefrontal and parietal regions in spatial working memory and problem-solving [48].

Our results also suggested the relationship between MoCA-AIS and spindle in the IFGtri (Figure 9). This finding is generally consistent with a recent study in children with attention deficit-hyperactivity disorder, which has shown the correlation between attention function and sleep spindle EEG activity in frontal [49]. In addition, a previous study in patients with MCI/mild dementia caused by Alzheimer's disease has shown that high-frequency repetitive transcranial magnetic stimulation in the right IFG significantly improved the attention and psychomotor speed [50]. On the other hand, in mouse models of epileptic encephalopathy Dravet syndrome and attention deficit disorder, decreased SK2 channel function in TRN has been observed, which is related to the generation of spindle action potential bursts [13]. Besides that, genes implied in attention disorders are expressed in sleep spindle-generating TRN circuits [51]. Attention involves concealed changes in neural representations of sensory stimuli without apparent behavioral changes, such as head or eye movements. Due to the fact that at any given moment, there is a large amount of sensory information in the external environment, far beyond the processing power in the brain, the human body must make adaptive decisions by means of suitably filtrating sensory input, enhancing related signals, and suppressing noise. The attention mechanism allows access to limited neural resources to select a small part of this information. A recent study by Nakajima et al. found a new pathway for attentional filtering, a prefrontal-basal ganglia-thalamus pathway that controls inhibition in the sensory thalamus [52]. This pathway enables sensory selection by suppressing distracting modality, enhances sensory discrimination, and contributes to goal-directed background noise suppression [52].

However, the contribution of spindle dysfunction to epilepsy and its underlying mechanisms are not yet well understood. One possibility is that the altered spindle activity may reflect neurotransmitter abnormalities and impaired synaptic plasticity, which are widely considered the main features of the disorder. Many studies suggest that epilepsy may result from an imbalance in glutamatergic and GABAergic neurotransmitter systems. The generating mechanisms of spindles also involve a feedback loop regulated primarily by GABAergic and glutamatergic neurotransmitters [13]. An example of the glutamatergic neurotransmitter is the AMPA receptor GluA1 subunit, coded by the GRIA1 locus. With regard to temporal lobe epilepsy, early studies reported altered GluA1 expression the hippocampus [53]. Notably, mice lacking the GluA1 subunit exhibited abnormal sleep EEG, reduced spindle activity [54], and impaired hippocampal synaptic plasticity [55, 56]. These findings suggest that glutamatergic dysfunction may play an important role in spindle disruption in patients with epilepsy.

These observations may contribute to a better understanding of the role of cortical thalamic circuits in the pathophysiology of epilepsy, enriching the relationship between regional changes in sleep spindle characteristics and specific cognitive domains impairment in neurological disorders, and exploring novel interventions. A growing body of evidence supports the dysfunction of thalamocortical circuits in epilepsy [4, 10]. The thalamus belongs to a subcortical structure, essential to synchronizing oscillations in the cortex [57]. Sleep spindles generated from TRN are dependent on interactions between the thalamus and cortex, which are at the core of cognition [13]. As a stable and inheritable signature of sleep EEG, sleep spindles may reflect the strength and malleability of cortical-thalamic circuits [13]. The spindle deficiency may arise from subtle changes in thalamocortical or intracortical circuits. Recent studies indicate that the connection strength of corticothalamic and thalamocortical would affect spindle characteristics. These stronger connectivity strengths are associated with relatively high spontaneous spindle density but have no significant influence on spindle synchronization [58]. Thalamocortical projections have two pathways [59]. The core thalamocortical system performs relatively strong and focal connections. In contrast, the matrix system is relatively weak and broad [58]. The regional differences in these connections may contribute to the complex temporal and spatial evolution of sleep spindle and nonuniform characteristics at the cortical level [58]. Moreover, one recent study that reported the disease course during six years of following-up of a child with epileptic encephalopathy has found a significant increase of spindles with high-dose diazepam therapy correlated with cognitive recovery, to some extent suggesting potential therapeutic targets of cognitive impairment [60]. However, more evidence about the therapeutic effect awaits further validation in future studies. Furthermore, a similar phenomenon of the altered spindle has also been observed in other brain disorders, such as neurodegenerative diseases (e.g., Parkinson's disease [61, 62] and Alzheimer's disease [39]) and psychiatric disorders (e.g., schizophrenia [63] and autism [64]). Sleep abnormalities are also firmly observed both in neurological and psychiatric disorders. The findings may indicate general trends of brain development and the aging process in brain disorders associated with sleep abnormalities rather than specific pathologic processes of epilepsy.

Sleep has a profound impact on epilepsy. A recent study showed the reduced number of sleep spindles before several minutes of seizure onset by comparing sleep spindle density in seizure night and control night, which supported the view that epileptic networks change ahead of seizure onset [65]. In addition, epilepsy patients develop sleep architecture abnormalities, including sleep disruption and instability, decreased REM sleep percentage, increased number of awakenings, and more sleep stage shifts [5]. The sigma power oscillates with a periodicity of approximately 0.02 Hz, dividing NREM sleep into spindle-poor and spindle-enriched periods, consistent with different levels of sensory arousals; for example, periods of spindle enrichment can prevent arousal and a spindle-poor period with a low arousal threshold [13]. The 0.02 Hz oscillation of sigma power balances two fundamental but opposing needs, maintaining sensory responsiveness to the environment while promoting recovery and memory consolidation, but perturbed 0.02 Hz oscillation may lead to memory impairment and ill-timed arousals in sleep disorders [66]. Sleep spindles play a critical role in sleep maintenance [13, 66], so spindle deficiency may contribute to understanding the increased sleep fragmentation in epilepsy patients.

Moreover, spindle activity varies with age and gender [15], so we included both age and gender as obligatory confounders in our multiple linear regression models. Another relevant factor affecting the sleep spindle is cardiac interference in EEG signals [15]. ECG artifacts are usually associated with body shape, and people with weight problems are more likely to present with cardiac interference. A recent study has found a negative correlation between spindle density and BMI by investigating sleep spindle characteristics in 11,630 individuals [15]. Since the typical duration of QRS complexes in ECG is approximately within the sigma range, the cardiac interference in EEG signals may increase the spindle detection threshold and decrease the number of the detected spindle [15]. We excluded individuals with BMI ≥ 28 kg/m^2^ in this analysis to reduce this effect.

However, our study has several limitations. A limitation of this study is that we did not consider the possible effect of epileptiform spikes during spindle analysis, and another is the lack of a healthy control group. Follow-up of our findings will be needed to better understand the effects of these changes on sleep spindle parameters. Another limitation of the available studies is that, in the description of cerebral locations named with corresponding craniocerebral anatomical regions, the error factors were possibly caused by the individual differences in the gross structures of the brain and scalp, such as F3/F4 electrodes (middle frontal gyrus) and F7/F8 electrodes (pars triangularis of inferior frontal gyrus). Due to the limited spatial resolution of the scalp EEG signal recording technique and the biological filtering effect in the scalp and skull, the recordings and measurements of the sleep spindle through this technique had an inherent limitation. Future studies may require combining other neuroimaging techniques for further exploration, for example, magnetoencephalography, intracranial local electrical recording technology, and event-related functional magnetic resonance imaging. Moreover, participants did not have a familiarization night. It was uncertain whether the first-night effect may affect the results or not. A sleep EEG of two nights or more would be recommended to increase the reliability of the data. This study is a cross-sectional and observational study design that cannot infer causality. It is unclear whether the changes in the sleep spindle are before the onset of clinical manifestations of cognitive impairment or a consequence of brain pathology. Although we found the altered spindle activity (density and duration) in the severe cognitive impairment subgroup compared to no/mild cognitive impairment subgroups, the small size of the severe cognitive impairment subgroup is another limitation of the current analysis that warrants consideration. Larger studies would better elucidate the between-group difference in spindle activity and the association between regional changes in sleep spindle and cognitive domain impairments. Of course, more longitudinal studies would also better assess the effectiveness of sleep spindle as a predictive biomarker for cognitive decline. An in-depth understanding of the relationship between sleep and cognition could ultimately contribute to the diagnosis and new therapeutic strategies.

## 5. Conclusions

In conclusion, this study suggested altered sleep spindle activity in epilepsy with severe cognitive impairment, compared with no/mild cognitive impairment. In multiple regression analysis models, we found the associations between the global cognitive status of adult epilepsy and spindle characteristics, and specific cognitive domains may relate to spindle characteristics in particular brain regions. The link between sleep spindles and epilepsy-related cognitive impairment may reflect the dysfunction in thalamic-reticular and thalamocortical mechanisms and could represent a potential biological marker of illness. Our cross-sectional and observational study design cannot infer causality. More longitudinal studies would be required to further assess the effectiveness of the sleep spindle as a biomarker for predicting cognitive decline.

## Figures and Tables

**Table 1:** Demographic and clinical characteristics and sleep architecture variables of participants.

Variables	Values
Age (years; mean (SD))	27.18 (6.11)
Female (*n* (%))	33 (57.9)
Handedness	Right
Education (years; mean (SD))	10.88 (4.56)
Age of onset (years; mean (SD))	17.65 (8.56)
Epilepsy duration (years; mean (SD))	9.56 (7.34)
Seizure type	
Partial (*n* (%))	4 (7.0)
Secondarily generalized (*n* (%))	31 (54.4)
Generalized (*n* (%))	22 (38.6)
Seizure frequency	
Less than once a year (*n* (%))	29 (50.9)
At least once a year (*n* (%))	17 (29.8)
At least once a month (*n* (%))	9 (15.8)
At least once a week (*n* (%))	2 (3.5)
NHS3 score (mean (SD))	12.30 (3.72)
Number of antiepileptic drugs (mean (SD))	1.11 (0.82)
History of febrile seizures (*n* (%))	14 (24.6)
Family history of epilepsy (*n* (%))	3 (5.3)
Waist-to-hip ratio (mean (SD))	0.81 (0.06)
Body mass index (kg/m^2^; mean (SD))	21.81 (3.08)
SAS score (mean (SD))	37.84 (9.31)
SDS score (mean (SD))	40.68 (10.82)
PSQI score (mean (SD))	3.91 (2.84)
Cognitive performance	
MMSE (mean (SD))	26.11 (4.09)
MoCA-TS (mean (SD))	22.07 (5.80)
MoCA-MIS (mean (SD))	10.74 (3.83)
MoCA-EIS (mean (SD))	8.84 (3.16)
MoCA-VIS (mean (SD))	4.96 (1.87)
MoCA-LIS (mean (SD))	4.39 (1.58)
MoCA-AIS (mean (SD))	14.16 (3.76)
MoCA-OIS (mean (SD))	5.60 (0.86)
Sleep parameters	
Total sleep time (TST, min; mean (SD))	447.95 (97.70)
Sleep efficiency (%; mean (SD))	69.45 (15.22)
NREM sleep stage 1 (% of TST; mean (SD))	7.35 (4.57)
NREM sleep stage 2 (% of TST; mean (SD))	60.10 (10.05)
NREM sleep stage 3 (% of TST; mean (SD))	13.18 (8.51)
REM sleep (% of TST; mean (SD))	19.36 (7.40)

NHS3: National Hospital Seizure Severity Scale; SAS: Self-rating Anxiety Scale; SDS: Self-rating Depression Scale; PSQI: Pittsburgh Sleep Quality Index; MMSE: Mini-Mental State Examination total score; MoCA-TS: Montreal Cognitive Assessment total score; MoCA-MIS: Memory Index Score; MoCA-EIS: Executive Index Score; MoCA-VIS: Visuospatial Index Score; MoCA-LIS: Language Index Score; MoCA-AIS: Attention Index Score; MoCA-OIS: Orientation Index Score; NREM: nonrapid eye movement; REM: rapid eye movement; SD: standard deviation.

**Table 2:** The results of bivariate correlation analysis.

Cognition	Education		Duration		Age of onset		NHS3 score		AEDs		WHR	
	*r*	*P*	*r*	*P*	*r*	*P*	*r*	*P*	*r*	*P*	*r*	*P*
MMSE	0.615	<0.001^∗^	-0.331	0.012^∗^	0.254	0.056	-0.178	0.185	-0.089	0.511	0.011	0.937
MoCA-TS	0.697	<0.001^∗^	-0.219	0.102	0.142	0.292	-0.151	0.263	-0.167	0.213	-0.120	0.374
MoCA-MIS	0.554	<0.001^∗^	-0.181	0.178	0.084	0.533	-0.050	0.714	-0.117	0.388	-0.234	0.080
MoCA-EIS	0.710	<0.001^∗^	-0.203	0.130	0.152	0.259	-0.200	0.136	-0.201	0.133	-0.093	0.493
MoCA-VIS	0.657	<0.001^∗^	-0.243	0.068	0.168	0.213	-0.150	0.265	-0.091	0.501	-0.151	0.262
MoCA-LIS	0.617	<0.001^∗^	-0.212	0.113	0.129	0.338	-0.096	0.477	-0.087	0.518	-0.080	0.555
MoCA-AIS	0.611	<0.001^∗^	-0.251	0.059	0.131	0.330	-0.114	0.397	-0.127	0.345	-0.055	0.684
MoCA-OIS	0.414	0.001^∗^	-0.206	0.125	0.217	0.104	-0.257	0.054	-0.141	0.294	0.142	0.290

^∗^Significance at *P* < 0.05; *r*: Pearson's correlation coefficient; duration: epilepsy duration; NHS3: National Hospital Seizure Severity Scale; AEDs: the number of antiepileptic drugs; WHR: waist-to-hip ratio; MMSE: Mini-Mental State Examination total score; MoCA-TS: Montreal Cognitive Assessment total score; MoCA-MIS: Memory Index Score; MoCA-EIS: Executive Index Score; MoCA-VIS: Visuospatial Index Score; MoCA-LIS: Language Index Score; MoCA-AIS: Attention Index Score; MoCA-OIS: Orientation Index Score.

## Data Availability

The data used to support the findings of this study are available from the corresponding author upon reasonable request.

## Supplementary Materials

Supplementary Materials Table S1: the differences in participants' characteristics between cognitive subgroups for MMSE scores. Table S2: the differences in participants' characteristics between cognitive subgroups for MoCA scores. Table S3: multiple linear regression with MMSE as the dependent variable. Table S4: multiple linear regression with MoCA-TS as the dependent variable. Table S5: multiple linear regression with MoCA-MIS as the dependent variable. Table S6: multiple linear regression with MoCA-EIS as the dependent variable. Table S7: multiple linear regression with MoCA-VIS as the dependent variable. Table S8: multiple linear regression with MoCA-LIS as the dependent variable. Table S9: multiple linear regression with MoCA-AIS as the dependent variable.
