# Supplementary material for: Assessment of Cognitive Function with Sleep Spindle Characteristics in Adults with Epilepsy
Source: Neural Plast. 2023 Apr 17;2023:7768980. doi: 10.1155/2023/7768980 (PMC10125769; doi:10.1155/2023/7768980)
Supplement: Supplementary Materials — Table S1: the differences in participants' characteristics between cognitive subgroups for MMSE scores. Table S2: the differences in participants' characteristics between cognitive subgroups for MoCA scores. Table S3: multiple linear regression with MMSE as the dependent variable. Table S4: multiple linear regression with MoCA-TS as the dependent variable. Table S5: multiple linear regression with MoCA-MIS as the dependent variable. Table S6: multiple linear regression with MoCA-EIS as the dependent variable. Table S7: multiple linear regression with MoCA-VIS as the dependent variable. Table S8: multiple linear regression with MoCA-LIS as the dependent variable. Table S9: multiple linear regression with MoCA-AIS as the dependent variable. [file 7768980.f1.docx]

# Assessment of cognitive function with sleep spindle characteristics in adults with epilepsy

## Supplementary Materials

Table S1: The differences in participants' characteristics between cognitive subgroups for MMSE scores.

| Variables | None (n=36) | | Mild (n=12) | | Severe (n=9) | | *F* | *P* |
| --- | --- | --- | --- | --- | --- | --- | --- | --- |
|  | Mean | SD | Mean | SD | Mean | SD |  |  |
| Age | 27.42 | 5.94 | 28.33 | 6.69 | 24.67 | 6.04 | 1.001 | 0.374 |
| Education years | 9.94 | 4.59 | 12.42 | 4.72 | 12.56 | 3.47 | 2.130 | 0.129 |
| Age of onset | 18.89 | 9.07 | 16.33 | 7.98 | 14.44 | 6.67 | 1.157 | 0.322 |
| Epilepsy duration | 8.59 | 7.04 | 12.02 | 9.11 | 10.14 | 5.68 | 1.015 | 0.369 |
| NHS3 score | 12.28 | 3.77 | 12.75 | 4.65 | 11.78 | 2.05 | 0.172 | 0.842 |
| Sleep efficiency (%) | 68.99 | 15.38 | 68.58 | 16.11 | 72.42 | 14.73 | 0.202 | 0.818 |
| NREM sleep stage 1 (%) | 7.51 | 4.06 | 8.23 | 6.87 | 5.53 | 2.05 | 0.958 | 0.390 |
| NREM sleep stage 2 (%) | 60.22 | 9.71 | 58.61 | 12.74 | 61.62 | 8.03 | 0.232 | 0.794 |
| NREM sleep stage 3 (%) | 13.44 | 9.29 | 12.43 | 7.08 | 13.19 | 7.71 | 0.061 | 0.940 |
| REM sleep (%) | 18.83 | 6.95 | 20.73 | 9.68 | 19.66 | 6.24 | 0.299 | 0.742 |

All variables meet homogeneity of variance assumptions based on Levene's test. No significant difference between subgroups by one-way ANOVA (all *P* > 0.05). The cognition grouping for Mini-Mental State Examination (MMSE) scores includes no cognitive impairment (≥ 27 points), mild cognitive impairment (23-26 points), and severe cognitive impairment (< 23 points). NHS3 = National Hospital seizure severity scale, NREM = non-rapid eye movement, REM = rapid eye movement.

Table S2: The differences in participants' characteristics between cognitive subgroups for MoCA scores.

| Variables | None (n=24) | | Mild (n=20) | | Severe (n=13) | | *F* | *P* |
| --- | --- | --- | --- | --- | --- | --- | --- | --- |
|  | Mean | SD | Mean | SD | Mean | SD |  |  |
| Age | 27.29 | 5.53 | 28.55 | 6.85 | 24.85 | 5.70 | 1.479 | 0.237 |
| Education years | 9.83 | 4.16 | 11.85 | 5.00 | 11.31 | 4.52 | 1.148 | 0.325 |
| Age of onset | 18.63 | 8.60 | 18.55 | 9.47 | 14.46 | 6.64 | 1.175 | 0.316 |
| Epilepsy duration | 8.69 | 7.09 | 10.09 | 8.12 | 10.34 | 6.93 | 0.285 | 0.753 |
| NHS3 score | 12.17 | 3.25 | 12.15 | 4.52 | 12.77 | 3.40 | 0.131 | 0.877 |
| Sleep efficiency (%) | 67.75 | 16.14 | 71.90 | 12.59 | 68.81 | 17.73 | 0.412 | 0.664 |
| NREM sleep stage 1 (%) | 7.17 | 3.49 | 7.29 | 4.39 | 7.79 | 6.58 | 0.079 | 0.925 |
| NREM sleep stage 2 (%) | 59.42 | 10.28 | 62.09 | 8.56 | 58.32 | 11.88 | 0.642 | 0.530 |
| NREM sleep stage 3 (%) | 14.49 | 10.34 | 11.66 | 6.13 | 13.12 | 8.17 | 0.594 | 0.556 |
| REM sleep (%) | 18.93 | 6.57 | 18.97 | 7.05 | 20.77 | 9.55 | 0.297 | 0.745 |

All variables meet homogeneity of variance assumptions based on Levene's test. No significant difference between subgroups by one-way ANOVA (all *P* > 0.05). The cognition grouping for Montreal Cognitive Assessment (MoCA) scores includes no cognitive impairment (≥ 25 points), mild cognitive impairment (20-24 points), and severe cognitive impairment (< 20 points). NHS3 = National Hospital seizure severity scale, NREM = non-rapid eye movement, REM = rapid eye movement.

Table S3: Multiple linear regression with MMSE as the dependent variable.

| Brain regions | Sleep spindle density | | | Sleep spindle duration | | |
| --- | --- | --- | --- | --- | --- | --- |
|  | *B* (95% CI) | *P* | *P*.adjust | *B* (95% CI) | *P* | *P*.adjust |
| Central | 0.104 (-0.104, 0.312) | 0.320 | 0.518 | -0.055 (-0.257, 0.147) | 0.586 | 0.763 |
| Middle frontal gyrus | -0.011 (-0.217, 0.194) | 0.914 | 0.960 | 0.025 (-0.172, 0.222) | 0.799 | 0.895 |
| IFGtri | 0.253 (0.051, 0.455) | 0 .015* | **0.074** | -0.262 (-0.435, -0.088) | 0.004* | **0.030** |
| Frontal pole | 0.132 (-0.061, 0.325) | 0.176 | 0.331 | -0.066 (-0.271, 0.140) | 0.523 | 0.713 |
| Occipital | 0.079 (-0.138, 0.295) | 0.469 | 0.670 | -0.019 (-0.235, 0.198) | 0.864 | 0.934 |
| Parietal | 0.103 (-0.106, 0.312) | 0.328 | 0.527 | -0.114 (-0.311, 0.083) | 0.250 | 0.428 |
| Middle temporal | 0.124 (-0.081, 0.330) | 0.230 | 0.403 | -0.093 (-0.295, 0.109) | 0.360 | 0.560 |
| Posterior temporal | 0.122 (-0.104, 0.347) | 0.283 | 0.473 | -0.065 (-0.290, 0.159) | 0.562 | 0.748 |
| Central midline | -0.064 (-0.271, 0.142) | 0.536 | 0.726 | 0.093 (-0.102, 0.287) | 0.344 | 0.543 |
| Frontal midline | 0.060 (-0.147, 0.268) | 0.560 | 0.746 | -0.023 (-0.242, 0.195) | 0.830 | 0.912 |
| Parietal midline | 0.041 (-0.155, 0.236) | 0.677 | 0.825 | -0.036 (-0.228, 0.156) | 0.708 | 0.840 |

Multiple regression models take the Z-score of Mini-Mental State Examination total score (MMSE) as the dependent variable, the Z-score of sleep spindle characteristic (density or duration) in each brain region as the independent variable. The covariates include age, gender, years of education, seizure frequency, epilepsy duration, and history of febrile seizure. For regression model, IFGtri = pars triangularis of inferior frontal gyrus, CI = confidence interval, * Statistical significance at *P* < 0.05 (uncorrected). After *P*-value correction via Benjamini & Hochberg method, the values marked in **bold** indicate significance at *P*.adjust < 0.10 (corrected).

Table S4: Multiple linear regression with MoCA-TS as the dependent variable.

| Brain regions | Sleep spindle density | | | Sleep spindle duration | | |
| --- | --- | --- | --- | --- | --- | --- |
|  | *B* (95% CI) | *P* | *P*.adjust | *B* (95% CI) | *P* | *P*.adjust |
| Central | 0.102 (-0.110, 0.315) | 0.336 | 0.535 | -0.099 (-0.303, 0.105) | 0.335 | 0.535 |
| Middle frontal gyrus | -0.002 (-0.214, 0.210) | 0.988 | 0.994 | 0.031 (-0.169, 0.231) | 0.758 | 0.872 |
| IFGtri | 0.217 (0.005, 0.430) | 0.045* | 0.159 | -0.246 (-0.430, -0.062) | 0.010* | **0.055** |
| Frontal pole | 0.157 (-0.039, 0.353) | 0.113 | 0.274 | -0.043 (-0.248, 0.162) | 0.674 | 0.825 |
| Occipital | 0.131 (-0.087, 0.348) | 0.233 | 0.407 | -0.121 (-0.340, 0.098) | 0.274 | 0.460 |
| Parietal | 0.227 (0.031, 0.424) | 0.024* | 0.103 | -0.213 (-0.405, -0.020) | 0.031* | 0.120 |
| Middle temporal | 0.162 (-0.045, 0.370) | 0.123 | 0.284 | -0.158 (-0.362, 0.046) | 0.126 | 0.286 |
| Posterior temporal | 0.123 (-0.109, 0.354) | 0.291 | 0.482 | -0.074 (-0.302, 0.154) | 0.519 | 0.712 |
| Central midline | -0.039 (-0.250, 0.171) | 0.708 | 0.839 | 0.084 (-0.117, 0.286) | 0.405 | 0.607 |
| Frontal midline | 0.114 (-0.099, 0.326) | 0.287 | 0.477 | -0.102 (-0.326, 0.122) | 0.364 | 0.564 |
| Parietal midline | 0.119 (-0.076, 0.314) | 0.227 | 0.398 | -0.126 (-0.318, 0.066) | 0.193 | 0.354 |

Multiple regression models take the Z-score of Montreal Cognitive Assessment total score (MoCA-TS) as the dependent variable, the Z-score of sleep spindle characteristic (density or duration) in each brain region as the independent variable. The covariates include age, gender, years of education, and seizure frequency. For regression model, IFGtri = pars triangularis of inferior frontal gyrus, CI = confidence interval, * Statistical significance at *P* < 0.05 (uncorrected). After *P*-value correction via Benjamini & Hochberg method, the values marked in **bold** indicate significance at *P*.adjust < 0.10 (corrected).

Table S5: Multiple linear regression with MoCA-MIS as the dependent variable.

| Brain regions | Sleep spindle density | | | Sleep spindle duration | | |
| --- | --- | --- | --- | --- | --- | --- |
|  | *B* (95% CI) | *P* | *P*.adjust | *B* (95% CI) | *P* | *P*.adjust |
| Central | 0.028 (-0.210, 0.266) | 0.816 | 0.903 | 0.017 (-0.212, 0.245) | 0.884 | 0.944 |
| Middle frontal gyrus | 0.010 (-0.226, 0.246) | 0.932 | 0.967 | 0.030 (-0.192, 0.252) | 0.786 | 0.890 |
| IFGtri | 0.135 (-0.108, 0.378) | 0.270 | 0.455 | -0.228 (-0.437, -0.019) | 0.033* | 0.125 |
| Frontal pole | 0.087 (-0.135, 0.309) | 0.437 | 0.638 | -0.041 (-0.269, 0.187) | 0.719 | 0.845 |
| Occipital | 0.111 (-0.132, 0.354) | 0.362 | 0.562 | -0.130 (-0.374, 0.114) | 0.289 | 0.479 |
| Parietal | 0.147 (-0.080, 0.373) | 0.199 | 0.360 | -0.145 (-0.366, 0.076) | 0.193 | 0.353 |
| Middle temporal | 0.180 (-0.051, 0.411) | 0.124 | 0.285 | -0.224 (-0.447, -0.001) | 0.049* | 0.167 |
| Posterior temporal | 0.034 (-0.226, 0.294) | 0.793 | 0.892 | -0.020 (-0.275, 0.234) | 0.875 | 0.939 |
| Central midline | -0.087 (-0.320, 0.145) | 0.455 | 0.655 | 0.100 (-0.124, 0.324) | 0.373 | 0.574 |
| Frontal midline | 0.056 (-0.182, 0.294) | 0.637 | 0.800 | -0.012 (-0.263, 0.239) | 0.924 | 0.964 |
| Parietal midline | 0.082 (-0.137, 0.301) | 0.455 | 0.654 | -0.080 (-0.296, 0.135) | 0.457 | 0.656 |

Multiple regression models take the Z-score of Memory Index Score (MoCA-MIS) as the dependent variable, the Z-score of sleep spindle characteristic (density or duration) in each brain region as the independent variable. The covariates include age, gender, years of education, and seizure frequency. For regression model, IFGtri = pars triangularis of inferior frontal gyrus, CI = confidence interval, * Statistical significance at *P* < 0.05 (uncorrected). After *P*-value correction via Benjamini & Hochberg method, none meets the significance at *P*.adjust < 0.10 (corrected).

Table S6: Multiple linear regression with MoCA-EIS as the dependent variable.

| Brain regions | Sleep spindle density | | | Sleep spindle duration | | |
| --- | --- | --- | --- | --- | --- | --- |
|  | *B* (95% CI) | *P* | *P*.adjust | *B* (95% CI) | *P* | *P*.adjust |
| Central | 0.104 (-0.096, 0.304) | 0.299 | 0.491 | -0.114 (-0.306, 0.077) | 0.237 | 0.413 |
| Middle frontal gyrus | 0.023 (-0.177, 0.223) | 0.816 | 0.903 | -0.003 (-0.192, 0.186) | 0.971 | 0.986 |
| IFGtri | 0.238 (0.041, 0.435) | 0.019* | **0.087** | -0.202 (-0.379, -0.025) | 0.026* | 0.109 |
| Frontal pole | 0.144 (-0.041, 0.329) | 0.125 | 0.285 | -0.068 (-0.261, 0.125) | 0.482 | 0.682 |
| Occipital | 0.117 (-0.089, 0.322) | 0.259 | 0.439 | -0.099 (-0.306, 0.109) | 0.344 | 0.542 |
| Parietal | 0.227 (0.042, 0.411) | 0.017* | **0.082** | -0.230 (-0.409, -0.051) | 0.013* | **0.065** |
| Middle temporal | 0.104 (-0.094, 0.303) | 0.296 | 0.488 | -0.087 (-0.283, 0.109) | 0.376 | 0.576 |
| Posterior temporal | 0.150 (-0.067, 0.367) | 0.172 | 0.326 | -0.076 (-0.291, 0.139) | 0.483 | 0.682 |
| Central midline | -0.001 (-0.200, 0.197) | 0.988 | 0.993 | 0.063 (-0.128, 0.254) | 0.511 | 0.704 |
| Frontal midline | 0.101 (-0.099, 0.302) | 0.316 | 0.513 | -0.130 (-0.340, 0.080) | 0.220 | 0.389 |
| Parietal midline | 0.135 (-0.048, 0.318) | 0.146 | 0.301 | -0.139 (-0.319, 0.041) | 0.128 | 0.287 |

Multiple regression models take the Z-score of Executive Index Score (MoCA-EIS) as the dependent variable, the Z-score of sleep spindle characteristic (density or duration) in each brain region as the independent variable. The covariates include age, gender, years of education, and seizure frequency. For regression model, IFGtri = pars triangularis of inferior frontal gyrus, CI = confidence interval, * Statistical significance at *P* < 0.05 (uncorrected). After *P*-value correction via Benjamini & Hochberg method, the values marked in **bold** indicate significance at *P*.adjust < 0.10 (corrected).

Table S7: Multiple linear regression with MoCA-VIS as the dependent variable.

| Brain regions | Sleep spindle density | | | Sleep spindle duration | | |
| --- | --- | --- | --- | --- | --- | --- |
|  | *B* (95% CI) | *P* | *P*.adjust | *B* (95% CI) | *P* | *P*.adjust |
| Central | 0.167 (-0.051, 0.384) | 0.130 | 0.287 | -0.145 (-0.355, 0.065) | 0.171 | 0.325 |
| Middle frontal gyrus | 0.061 (-0.158, 0.281) | 0.577 | 0.755 | -0.054 (-0.262, 0.153) | 0.602 | 0.778 |
| IFGtri | 0.097 (-0.131, 0.325) | 0.399 | 0.603 | -0.088 (-0.291, 0.115) | 0.387 | 0.590 |
| Frontal pole | 0.136 (-0.070, 0.341) | 0.190 | 0.350 | 0.030 (-0.184, 0.243) | 0.781 | 0.889 |
| Occipital | 0.108 (-0.119, 0.335) | 0.345 | 0.543 | -0.077 (-0.307, 0.152) | 0.501 | 0.694 |
| Parietal | 0.222 (0.016, 0.427) | 0.035* | 0.131 | -0.120 (-0.327, 0.087) | 0.250 | 0.428 |
| Middle temporal | 0.113 (-0.106, 0.332) | 0.304 | 0.497 | -0.081 (-0.297, 0.135) | 0.457 | 0.655 |
| Posterior temporal | 0.042 (-0.201, 0.285) | 0.732 | 0.853 | 0.002 (-0.236, 0.240) | 0.984 | 0.991 |
| Central midline | -0.062 (-0.280, 0.156) | 0.569 | 0.751 | 0.128 (-0.079, 0.336) | 0.220 | 0.389 |
| Frontal midline | 0.068 (-0.154, 0.290) | 0.540 | 0.728 | -0.092 (-0.325, 0.142) | 0.433 | 0.633 |
| Parietal midline | 0.128 (-0.075, 0.331) | 0.211 | 0.376 | -0.174 (-0.371, 0.022) | 0.081^#^ | 0.241 |

Multiple regression models take the Z-score of Visuospatial Index Score (MoCA-VIS) as the dependent variable, the Z-score of sleep spindle characteristic (density or duration) in each brain region as the independent variable. The covariates include age, gender, years of education, and seizure frequency. For regression model, IFGtri = pars triangularis of inferior frontal gyrus, CI = confidence interval, * Statistical significance at *P* < 0.05 (uncorrected), ^#^ Statistical trend at *P* < 0.10 (uncorrected). After *P*-value correction via Benjamini & Hochberg method, none meets the significance at *P*.adjust < 0.10 (corrected).

Table S8: Multiple linear regression with MoCA-LIS as the dependent variable.

| Brain regions | Sleep spindle density | | | Sleep spindle duration | | |
| --- | --- | --- | --- | --- | --- | --- |
|  | *B* (95% CI) | *P* | *P*.adjust | *B* (95% CI) | *P* | *P*.adjust |
| Central | 0.172 (-0.046, 0.389) | 0.119 | 0.279 | -0.157 (-0.366, 0.053) | 0.139 | 0.294 |
| Middle frontal gyrus | 0.104 (-0.114, 0.323) | 0.342 | 0.540 | -0.090 (-0.297, 0.117) | 0.388 | 0.590 |
| IFGtri | 0.189 (-0.035, 0.413) | 0.097^#^ | 0.265 | -0.148 (-0.348, 0.053) | 0.146 | 0.300 |
| Frontal pole | 0.226 (0.027, 0.425) | 0.027* | 0.111 | -0.068 (-0.281, 0.145) | 0.524 | 0.713 |
| Occipital | 0.156 (-0.069, 0.382) | 0.170 | 0.324 | -0.178 (-0.403, 0.047) | 0.119 | 0.279 |
| Parietal | 0.194 (-0.015, 0.402) | 0.068^#^ | 0.213 | -0.150 (-0.357, 0.056) | 0.149 | 0.302 |
| Middle temporal | 0.170 (-0.046, 0.386) | 0.120 | 0.280 | -0.099 (-0.315, 0.117) | 0.361 | 0.561 |
| Posterior temporal | 0.143 (-0.097, 0.384) | 0.237 | 0.412 | -0.047 (-0.285, 0.191) | 0.692 | 0.830 |
| Central midline | 0.077 (-0.142, 0.295) | 0.484 | 0.682 | -0.022 (-0.233, 0.190) | 0.838 | 0.916 |
| Frontal midline | 0.205 (-0.011, 0.420) | 0.063^#^ | 0.202 | -0.134 (-0.366, 0.098) | 0.253 | 0.432 |
| Parietal midline | 0.128 (-0.075, 0.332) | 0.211 | 0.375 | -0.170 (-0.367, 0.028) | 0.091^#^ | 0.255 |

Multiple regression models take the Z-score of Language Index Score (MoCA-LIS) as the dependent variable, the Z-score of sleep spindle characteristic (density or duration) in each brain region as the independent variable. The covariates include age, gender, years of education, and seizure frequency. For regression model, IFGtri = pars triangularis of inferior frontal gyrus, CI = confidence interval, * Statistical significance at *P* < 0.05 (uncorrected), ^#^ Statistical trend at *P* < 0.10 (uncorrected). After *P*-value correction via Benjamini & Hochberg method, none meets the significance at *P*.adjust < 0.10 (corrected).

Table S9: Multiple linear regression with MoCA-AIS as the dependent variable.

| Brain regions | Sleep spindle density | | | Sleep spindle duration | | |
| --- | --- | --- | --- | --- | --- | --- |
|  | *B* (95% CI) | *P* | *P*.adjust | *B* (95% CI) | *P* | *P*.adjust |
| Central | 0.101 (-0.116, 0.318) | 0.355 | 0.557 | -0.064 (-0.274, 0.146) | 0.544 | 0.729 |
| Middle frontal gyrus | 0.091 (-0.124, 0.306) | 0.399 | 0.602 | -0.025 (-0.229, 0.180) | 0.810 | 0.901 |
| IFGtri | 0.208 (-0.010, 0.427) | 0.061^#^ | 0.197 | -0.233 (-0.423, -0.043) | 0.017* | **0.081** |
| Frontal pole | 0.142 (-0.060, 0.343) | 0.164 | 0.317 | -0.058 (-0.268, 0.151) | 0.580 | 0.757 |
| Occipital | 0.088 (-0.137, 0.312) | 0.437 | 0.638 | -0.121 (-0.345, 0.103) | 0.284 | 0.474 |
| Parietal | 0.187 (-0.018, 0.393) | 0.072^#^ | 0.222 | -0.212 (-0.409, -0.014) | 0.036* | 0.132 |
| Middle temporal | 0.097 (-0.119, 0.313) | 0.370 | 0.571 | -0.031 (-0.244, 0.183) | 0.775 | 0.885 |
| Posterior temporal | 0.148 (-0.088, 0.384) | 0.213 | 0.378 | -0.094 (-0.327, 0.139) | 0.421 | 0.623 |
| Central midline | 0.101 (-0.113, 0.314) | 0.347 | 0.545 | -0.063 (-0.270, 0.143) | 0.540 | 0.727 |
| Frontal midline | 0.157 (-0.058, 0.372) | 0.149 | 0.302 | -0.094 (-0.324, 0.135) | 0.413 | 0.614 |
| Parietal midline | 0.147 (-0.051, 0.345) | 0.143 | 0.298 | -0.132 (-0.328, 0.064) | 0.182 | 0.338 |

Multiple regression models take the Z-score of Attention Index Score (MoCA-AIS) as the dependent variable, the Z-score of sleep spindle characteristic (density or duration) in each brain region as the independent variable. The covariates include age, gender, years of education, and seizure frequency. For regression model, IFGtri = pars triangularis of inferior frontal gyrus, CI = confidence interval, * Statistical significance at *P* < 0.05 (uncorrected), ^#^ Statistical trend at *P* < 0.10 (uncorrected). After *P*-value correction via Benjamini & Hochberg method, the values marked in **bold** indicate significance at *P*.adjust < 0.10 (corrected).
